# Exploring the interaction between extracellular matrix components in a 3D organoid disease model to replicate the pathophysiology of breast cancer

**DOI:** 10.1186/s13046-023-02926-4

**Published:** 2023-12-16

**Authors:** Anamitra Bhattacharya, Kamare Alam, Nakka Sharmila Roy, Kulwinder Kaur, Santanu Kaity, Velayutham Ravichandiran, Subhadeep Roy

**Affiliations:** 1 Department of Pharmacology and Toxicology, National Institute of Pharmaceutical Education and Research, Kolkata, West Bengal 700054 India; 2 School of Pharmacy and Biomolecular Sciences, RCSI University of Medicine a Health Sciences, Dublin, Ireland; 3 Tissue Engineering Research Group, Department of Anatomy & Regenerative Medicine, RCSI University of Medicine and Health Sciences, Dublin, Ireland

**Keywords:** 3D organoid, In-vitro disease model, Cell patterning, Tumor microenvironment, Extra cellular matrix, Cancer associated fibroblast, Breast cancer

## Abstract

In vitro models are necessary to study the pathophysiology of the disease and the development of effective, tailored treatment methods owing to the complexity and heterogeneity of breast cancer and the large population affected by it. The cellular connections and tumor microenvironments observed in vivo are often not recapitulated in conventional two-dimensional (2D) cell cultures. Therefore, developing 3D in vitro models that mimic the complex architecture and physiological circumstances of breast tumors is crucial for advancing our understanding of the illness. A 3D scaffold-free in vitro disease model mimics breast cancer pathophysiology by allowing cells to self-assemble/pattern into 3D structures, in contrast with other 3D models that rely on artificial scaffolds. It is possible that this model, whether applied to breast tumors using patient-derived primary cells (fibroblasts, endothelial cells, and cancer cells), can accurately replicate the observed heterogeneity. The complicated interactions between different cell types are modelled by integrating critical components of the tumor microenvironment, such as the extracellular matrix, vascular endothelial cells, and tumor growth factors. Tissue interactions, immune cell infiltration, and the effects of the milieu on drug resistance can be studied using this scaffold-free 3D model. The scaffold-free 3D in vitro disease model for mimicking tumor pathophysiology in breast cancer is a useful tool for studying the molecular basis of the disease, identifying new therapeutic targets, and evaluating treatment modalities. It provides a more physiologically appropriate high-throughput platform for screening large compound library in a 96–384 well format. We critically discussed the rapid development of personalized treatment strategies and accelerated drug screening platforms to close the gap between traditional 2D cell culture and in vivo investigations.

**Graphical Abstract:**

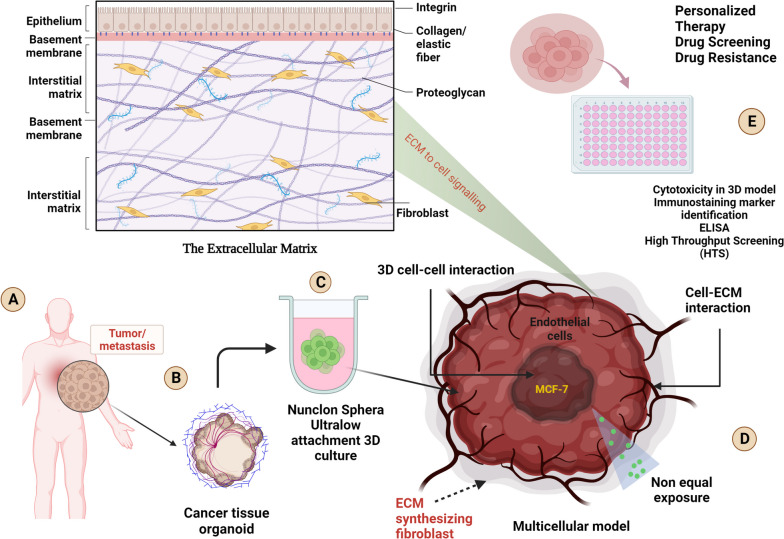

## Introduction

An organoid is a small, three-dimensional (3D) cell assembly that is heterocellular in nature. Primary organoids are composed of cells that come from donor tissues and can self-organize and change into different cell types to mimic the complex cellular organization and makeup of an organ. Organoids provide a more accurate picture of human health than traditional 2D cell culture and animal models. This makes them an important tool for biomedical research [1]. Organoids derived from human breast tissue are a unique way to study the biology and pathology of breast cancer in a manner that is controlled and specific to each patient. Personalized treatment and the study of how different tumors are from each other are both possible because of the primary breast cancer biopsies [2]. Organoids provide a more accurate picture of how a patient’s tumor works than standard 2D cell cultures or animal models, because they maintain the cell complexity and characteristics of the original tumor. Researchers can use organoids to look for drugs and measure how each patient responds to a certain course of treatment as a personalized therapy [3]. A 3D multicellular heterogenic scaffold created without the help of a scaffold material (synthetic, polymeric, or extracellular matrix) is referred to as a scaffold-free organoid. The traditional organoid production approach involves embedding cells within a gel-like matrix such as Matrigel or other cross-linkable/thermoresponsive hydrogels, which offer structural support for their development and growth. However, scaffold-free organoids allow cells to self-assemble and organize into complex structures without the use of an external scaffolding substance [4]. High consistency and reproducibility are the two significant benefits of scaffold-free organoid models. They enable the accurate measurement of cellular responses, such as extracellular matrix synthesis, without being hampered by exogenous collagen.

A scaffold or support structure is used to create a three-dimensional (3D) structure that resembles the native tissue. This structure is called a scaffold-based organoid, and it is stable and resembles the organ being studied or made [5].. To create scaffold-based organoids, cells are placed inside a material called a scaffold, which provides structural support, helps cells stick together, and makes it easier for tissue structures to form in an organized manner. Scaffolds can be fabricated from biomaterials such as hydrogels, polymers, and parts of the extracellular matrix (ECM). These supports provide cells with a three-dimensional structure for interacting with their surroundings and arranging themselves into structures that resemble tissues in the target organ. Organoids built on scaffolds have several advantages. First, it provides a good microenvironment that helps cells grow into different types of cells and form new tissues. It is possible to recreate the position of cells, presence of ECM components, and other important parts of the structure of the original tissue. Second, scaffold-based organoids can be prepared to include specific cell types or cells obtained from a patient. This makes it possible to use personalized medical methods and model diseases. Third, they can be used to study organ growth, physiology, and pathology in controlled laboratory settings. Depending on where and how they are used, scaffold-based organoids have different limitations. In general, the following are the problems with scaffold-based organoid systems: To help organoids grow and keep cells alive, the support must be biocompatible and have a certain structure. However, some scaffolds might need to work better with certain cell types or regions of interest, which could lead to growth or differentiation that is not as good as possible. Its biocompatibility should be carefully examined to ensure that its structure does not affect the cell lineage in organoids [6, 7]. Scaffolds are often made and prepared using complicated steps, and differences in the way scaffolds are made can affect the organoids, which in turn affects their reproducibility [8]. Scaffolds can make it harder for nutrients to penetrate through the organoid structure, which can affect the health of the cells and make the core areas hypoxic, nutrient deficient, and growth factor deficient. The thickness and porosity of the scaffold can affect how nutrients and release factors are distributed, which could affect how organoids grow and work as a whole. The phenotype and behavior of cells can be changed in response to different materials used. Sometimes, the scaffold can change the cellular lineage and affect differentiation, maturation, and signaling pathways. It is important to ensure that the scaffold has the same capabilities as those of natural tissues [9]. Although scaffold-based organoids try to mimic the structure of native tissues, they may not be able to fully replicate the complex structure and biological organization found in vivo. The scaffold could be used as a general structural support, but it might not be able to replicate the complex relationships between cells and their arrangement in natural organs [10].

In this review we have dive into the critical discussion of the following question.

- We have gone over the types of extracellular matrix components and how they work to keep the breast cancer microenvironment intact. Finding the right mix and selecting natural extracellular matrix components produced by specific cells over synthetic ones as scaffold materials is illuminating.
- The focus here is on the role of extracellular matrix (ECM) functions in organoid development and pathology, as well as the downstream signals mediated by their constituent parts.
- How cellular manipulation can be applied to produce its own required ECM in 3D Organoid by involving variable cell types (Fibroblast, HUVEC, Cancer Stem Cell) to develop scaffold free 3D organoid is discussed in details. Different cutting-edge technique to developed for organoid vascularization also covered.
- The role of the stromal component and the diverse immune cells (M1, M2) in breast organoid formation, as well as their interaction, are examined in terms of future prospects.

## Intermediate role of extracellular matrix in tumor microenvironment

The extracellular matrix (ECM) is an arrangement or network of extracellular macromolecules that structurally and biochemically supports neighboring cells. Cell adhesion, cell-to-cell communication, and differentiation are standard ECM functions. It is essential for many biological functions, including maintaining tissue integrity and controlling cell behavior. It is a scaffold for cells that enables their adhesion, migration, and interaction with their surroundings [11]. Native tissues and organs are sources of natural ECMs components. They comprise a complex network of proteins (e.g., collagen, fibronectin, laminin, and elastin) and polysaccharides (e.g., glycosaminoglycans). They serve as structural scaffolds and offer biochemical cues to support cellular activities. Researchers have attempted to make artificial or synthetic ECMs biocompatible and can communicate with cells via ligand-receptor interactions. However, their limited in vivo stability and susceptibility to enzymatic degradation restrict their long-term applications in tissue engineering [12].

### Natural extracellular matrix component

Collagen I and ECM modifiers regulate the stiffening and self-assembly of the cancer cell matrix, which is essential for breast cancer invasion, and the invasive expansion of new branches has been studied in mammary organoids grown from single primary human basal cells in 3D-collagen gels. Invasion implies a strict need for spatiotemporal regulation of ECM viscoelasticity and stiffness. Collagen remodeling during branch elongation has been observed in a separate study to be caused by collective cell migration occurring within the branch, which is characterized by a back-and-forth movement and tension balance between the branch and the surrounding matrix. Researchers have shown that a lack of sufficient elastic restoring forces and local yielding of the residual collagen matrix initiate the final stage of organoid formation [13, 14].

Collagen scaffolds allow for the development of highly spherical organoids that resemble normal human breast acini [15]. The scaffolds provide a suitable environment for the growth and organization of breast cancer cells, allowing the study of metastatic events and the induction of epithelial to mesenchymal transition (EMT) and mesenchymal to epithelial transition (MET) [16]. Collagen scaffolds can be designed with directional/anisotropic or nondirectional/isotropic porous architectures to modulate the migration rate of seeded cells and capture the detection of a migrated population within a set time [17]. Collagen-based scaffolds have drawbacks as breast scaffold materials because of their poor mechanical properties, which limits their applications to some extent [18]. Additionally, the characteristics of collagen scaffolds, such as the mean pore size and interconnectivity, can influence cellular responses and invasion into the scaffold. Although collagen scaffolds offer good permeability, biocompatibility, and biodegradability, they may not adequately replicate the tumor microenvironment in breast cancer research [19]. Overall, the drawbacks of collagen as a breast scaffold material include its poor mechanical properties and limitations in replicating the tumor microenvironment.

Epithelial biology relies on laminin 332, a large extracellular matrix protein composed of 332 subunits that helps maintain cell adhesion, polarity, proliferation, and differentiation. In addition, it aids in tissue development, maintenance, and growth. Tumor invasiveness is linked to the aberrant expression of laminin 332 [20].

Laminin-111 (LN1) has been shown to be indispensable for the formation of normal breast acini. In 3D culture models, laminin-derived peptides have been found to regulate gene and protein expression in breast cancer cells, including the expression of GPNMB, a protein associated with malignant phenotypes [21]. The presence of laminin in the extracellular matrix promotes cell attachment and viability, facilitating the self-organization of primary breast cancer cells into tumoroids [22]. Additionally, breast cancer stem cells produce a laminin matrix that promotes self-renewal and tumor initiation by engaging specific integrins and activating signaling pathways [23]. However, changes in ECM composition, such as the presence of laminin, can alter estrogen responsiveness and the effectiveness of antiestrogen therapies in estrogen receptor (ER)-positive breast cancer cells [24]. Another drawback is that breaks in the continuity of laminin occur in breast carcinomas and have been implicated in tumor metastasis. Additionally, laminin expression is significantly higher in breast cancer tissues than in normal breast tissues, suggesting its involvement in breast cancer invasion and metastasis [25].

Elastin-like polypeptides (ELPs) have been highlighted for their potential as adaptable and cost-effective platforms for spheroid culture, and their role in spheroid generation is discussed [26]. Using 3D in vitro cancer modeling, researchers have investigated whether Elastin-Like Recombinant (ELR) polypeptides are promising candidates for recreating breast cancer ECM. Two ELR polypeptides were used to create the hydrogels, one with cell adhesion motifs and the other with MMP-cleavage sites. It is currently unclear how ELRs with varying matrix stiffness and tumor-ECM motifs affect breast cancer cell invasion and progression. The role of ECM in breast cancer progression and medication response has not been fully elucidated, and the significance of this biomaterial in this regard remains obscure [27].

Elastin-based scaffolds have been explored as biomaterials for use in breast organoid models. Elastin-like recombinamer (ELR) hydrogels, composed of two ELR polypeptides, have shown promise in mimicking the extracellular matrix (ECM) of breast tumors and in supporting cell viability and proliferation [27]. Elastin-based hydrogels, formed by elastin-like recombinamers (ELRs), have demonstrated high viability and cell proliferation for up to 7 days when cultured with breast cancer or non-tumorigenic breast cells [4]. ELR hydrogels were used to culture MCF7 and MCF10A cells, which formed spheroids, and MDA-MB-231 cells, which formed cell networks [27]. Elastin has some drawbacks when used as a scaffold material. One of the limitations is their insolubility, which makes it difficult to process them into biomaterials [28]. Additionally, elastin has low ultimate tensile strength, which restricts its use as an arterial conduit [29]. Another challenge is that Elastin lacks a bioactive domain for cell adhesion, proliferation, and differentiation [30].

Role of fibronectin supplementation as a hydrogel extracellular matrix in regulating cell behavior at the biomaterial interface. FBN facilitates cell adherence, spreading, migration, proliferation, and differentiation [31] Fibronectin plays a crucial role in controlling how cells adhere to one another, disseminate, migrate, proliferate, differentiate, and discusses approaches to improve biomaterial surfaces with fibronectin. Protein conformational adsorption is highly substrate-dependent, making it difficult to exert complete control over the process during immobilization of the entire FBN in the hydrogel. However, anchoring of the FBN fragment is preferable for the immobilization of single binding domains, because proper interaction with cell integrins requires the interaction of several FBN-specific domains [32].

Novel tessellated three-dimensional polymer scaffolding induces an epithelial-mesenchymal transition (EMT)-like event through the production of a fibrillar fibronectin matrix [33]. ECM fibrillar components, including fibronectin, affect the behavior and properties of mammary cancer cells, thereby influencing their invasive potential [34]. Amyloid-fibril hydrogels, which mimic the extracellular matrix, provide a biomimetic ECM scaffold for 3D cell culture and tumor spheroid formation. These hydrogels support the formation of breast tumor spheroids with a well-defined necrotic core and cancer-associated gene expression, resembling the original tumor [35]. Fibronectin has been shown to have drawbacks when used as a breast scaffold material. High fibronectin expression is strongly associated with decreased patient survival, indicating a negative impact on prognosis [33]. Fibronectin can induce the expression of MMP-2, which is responsible for ECM degradation and tumor invasion. Additionally, fibronectin deposition and matrix mettalo proteinase (MMP) activation have been implicated in the regulation of tumor dormancy and subsequent outgrowth, leading to drug resistance and aggressive behavior [36]. Furthermore, degradation of fibronectin by MMP-9 can promote cell invasion and migration, potentially contributing to breast cancer progression [37].

Proteoglycans are complex molecules composed of linked chains of the main proteins and glycosaminoglycans (GAGs). ECM hydration, compression resistance, and signal transduction pathway regulation are bolstered by these molecules. Proteoglycans (PGs) play a crucial role in the expansion and spread of breast cancer and affect cell behavior and signaling [38, 39].

The biochemical composition of proteoglycans in breast tissues has been studied, and it was found that proteoglycans are more abundant in neoplastic tissues than in nonneoplastic tissues. Specifically, an increase in chondroitin sulfate and a decrease in dermatan sulfate were observed in tumors compared to benign lesions [40]. These changes in proteoglycans indicate significant alterations in the extracellular matrix and surface properties of cells in breast cancer tissues. Additionally, the interaction between cell-associated and tumor microenvironment glycosaminoglycans/proteoglycans and their roles in cancer pathogenesis and progression have been explored [41]. However, there are some drawbacks to their use. First, proteoglycans extracted from the breast tissues of patients with invasive mammary carcinoma or benign lesions showed significant changes in their composition compared to nonneoplastic tissues. Second, biochemical data indicated an increase in the overall proteoglycan content in tumors, suggesting that they may contribute to the progression of breast cancer [38].

ECM components are produced by a variety of cells in our bodies, including fibroblasts, indicating that by co-culturing human dermal fibroblasts (HDFs) and JIMT-1 human breast cancer cells in the presence of TGF-β, fibroblasts produce fibronectin, collagen I, and laminin. As a result of this research, we conclude that fibroblasts produce various natural ECM components that are required for cell adhesion, migration, differentiation, and cell-to-cell interactions in tumor formation. This study also promotes the utilization of fibroblasts rather than synthetic ECM for the development of scaffold-free 3D breast organoid structures [42].

### Synthetic polymeric ECM

Synthetic ECMs are engineered biomaterials that aim to imitate the properties of natural ECMs while avoiding their disadvantages. The content and properties of these matrices can be manipulated more precisely with the help of synthetic polymers and peptides. Synthetic ECMs offer greater stability in vivo, may be engineered to have variable mechanical properties, and provide functional peptide epitopes that influence cellular interactions. They aid in tissue regeneration and repair by facilitating cell adhesion, proliferation, migration, and differentiation [43].

Three-dimensional polymer networks, also known as hydrogels, can affect the properties of the extracellular matrix. They can be developed to possess certain physical, chemical, and biological properties, making them useful for tissue engineering and medication delivery. An article examined how biomaterials can be used to create synthetic ECMs for analyzing the adaptability of cancer. This highlights the role of biophysical cues in regulating cancer cell behavior, and explores their potential implications for research on carcinogenesis and personalized medicine [44]. Tumor organoids can be modified and an improved in vitro representation of ECM-regulated tumor growth can be achieved using hydrogels based on extracellular matrix components [45]. Synthetic peptides can imitate ECM sections and bind to cell surface receptors or ECM components. These peptides can modulate cellular responses, promote cell adhesion, and influence ECM remodeling. Researchers have investigated the role of adhesion signals in the microenvironment on the expansion of mammary epithelial cells (MEC) and the progression of breast cancer using synthetic hydrogels. Previous research has indicated that RGD and YIGSR, two adhesion peptides, regulate the formation of distinct phenotypes in malignant and non-malignant MECs [46]. This review discusses ECM fragments and their interactions with integrins under pathophysiological conditions [47]. To facilitate cell proliferation and tissue repair, synthetic polymers, such as polylactic-co-glycolic acid (PLGA) and polyethylene glycol (PEG), can be molded into porous scaffolds. These scaffolds have desirable properties such as a certain mechanical strength or degradation rate [48, 49].

Synthetic hydrogels have also been explored as scaffolds for breast organoids. Polyethylene glycol-derived hydrogels (PEG), gelatin methacryloyl (GelMA), and thiolated-gelatin crosslinked with PEG-4MAL (GelSH) have been successfully used to support the growth and organoid formation of breast cancer cells [50]. Polyisocyanide (PIC) hydrogels have also been developed as synthetic biomimetic matrices for mammary gland organoids (MGOs) [41]. Collagen I-blended agarose hydrogels have been shown to influence the growth, size, morphology, and motility of breast cancer cell spheroids [51]. Poly (lactic-co-glycolic) acid (PLGA) and polycaprolactone (PCL) have been used to fabricate porous scaffolds for breast cancer cells, which exhibit distinct survival, morphology, and proliferation compared with 2D cultures [52]. One major drawback is the reliance on poorly defined animal-derived extracellular matrices, which limits their application in regenerative and translational medicine [41]. Another drawback is the limited ability to customize and control the biophysical and biochemical parameters of the hydrogel matrix [53]. Additionally, synthetic hydrogels may not fully recapitulate the tissue-specific environment necessary for organoid growth and differentiation.

Synthetic peptide epitopes are amino acid sequences engineered to act similar to their natural ECM protein counterparts. By incorporating these peptides into biomaterials, biological interactions can be modulated. Natural and synthetic peptide epitopes are discussed as molecular tools for designing bioactive hydrogel materials for controlling cell-cell and cell-extracellular matrix interactions as well as cellular and tissue function, repair, and regeneration [54]. One-dimensional nanostructured templates made from peptide nanofibers have several potential applications in medicine and nanotechnology [55]. The interaction between the integrin receptors of fibroblasts and cell adhesion motifs of the scaffolds stimulates cell migration, similar to the natural extracellular matrix [56]. One of the main challenges associated with their use is the limited control over the ratio of cell types within the organoid, which is influenced by the interactions between the cells and peptide scaffold. Additionally, the stiffness of the peptide scaffold can affect the colony formation efficiency, indicating the importance of optimizing the mechanical properties of the scaffold [41]. Another concern is the potential cytotoxic effects of functionalized peptides on cells. For example, in one study, the cytotoxicity of a mineralized peptide scaffold was found to depend on the immobilization of the peptide on the scaffold [57].

Synthetic polymer scaffolds have been shown to support the survival, morphology, and proliferation of breast cancer cells as well as the expression of extracellular matrix proteins and their receptors in mammary epithelial cells. The hydrophobic nature of synthetic polymers can be a limitation for tissue engineering applications; however, hydrophilization techniques have been developed to improve cell/tissue compatibility within scaffolds [52].

**Fig. 1:**
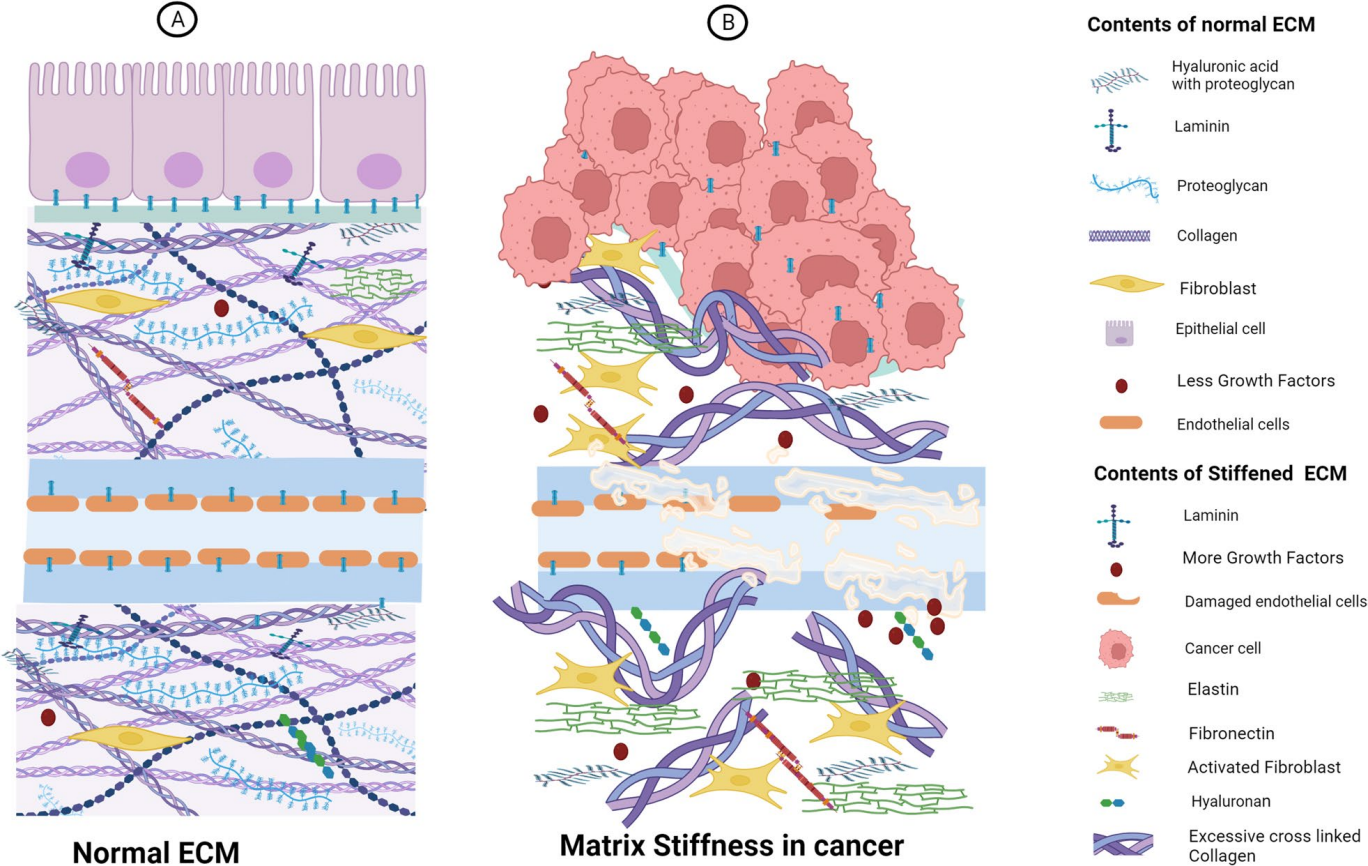
Schematic representation of variable Extra Cellular Matrix (ECM) components in healthy ECM and TME. **A** ECM components in healthy tissue (**B**) Matrix stiffness is mostly caused by an abundance of collagen and HA within the TME

Synthetic ECM describes artificially engineered matrices that resemble the structure and functionality of natural ECM. They provide exact control over matrix composition, mechanical characteristics, and biochemical signaling. The following are some drawbacks and benefits: It is possible that artificial ECM cannot fully replicate the complexity and diversity of the native ECM found in breast tissue owing to a lack of complexity. Bioactive components may be missing from synthetic matrices in the natural ECM and may affect cell behavior and tumor development [58, 59]. (Fig. 1) (Table 1).

**Table 1:** Intermediate role of extracellular matrix (ECM)

	Component	Advantages	Disadvantages	Ref.
Natural ECM	Collagen	Recapitulation of In-Vivo Microenvironment, Relevant ECM Composition, Mimicking Breast Cancer Stiffness, Patient-Specific Drug Testing	Lack of Complexity, Stiffness Variability, ECM Heterogeneity, Matrix Remodeling Challenges	[13, 60, 61]
	Elastin	Elasticity and Resilience, Biocompatibility and Biodegradability, No Immunogenicity	Limited Availability, Remodeling and Degradation, Influence on Tumor Growth	[26, 62, 63]
	Fibronectin	Scaffold for Tissue Development, Regulator of Cell Signal Transduction, Modulation of Cell Behavior, Potential Therapeutic Applications	Altered Cell Behavior, Potential Disease Implications, Limited Control of ECM Expression	[31, 32]
	Laminin	Biomimetic Nature, Cell Behavior Regulation, Maintenance of Epithelial Cohesion	Overexpression in Breast Cancer, Complexity in Processing, Spatial Control and Heterogeneity	[20, 64]
	Proteoglycans	Improved Biomimicry, Cellular Communication and Interaction, Matrix Turnover	ECM Complexity, Cost	[38, 39]
Synthetic ECM	Synthetic Hydrogels	Recreating Tumor Microenvironment, Cell-Cell and Cell-Matrix Interactions, Tissue Structure and Function, Alternative to Animal Models	Cost, Standardization and Reproducibility, Limited Availability of Synthetic Hydrogels, Learning Curve	[44, 45]
	Functionalized Peptides	Biomimicry, Cell-Cell and Cell-ECM Interactions, Tumor Microenvironment Modeling, Drug Screening Platforms	Cost, Lack of Standardization, Limited Peptide Repertoire	[46, 47]
	Synthetic Polymer Scaffolds	Mimicking Tumor Microenvironment, Improved Cell-Cell and Cell-Matrix Interactions, Drug Screening and Therapeutic Efficacy Evaluation, Biocompatibility	Complexity of Fabrication, Limited Representation of In Vivo Conditions, Interference with Cellular Signaling	[18, 49]
	Synthetic Peptide Epitopes	Mimicking Native Tumor Microenvironment, Regulating Biological Processes, Tunable 3D Models	Cost, Incomplete Replication of In-Vivo Environment, Lack of Long-term Stability	[54, 55]

## Functions of ECM in organoid development and pathophysiology

### Immunogenicity aspect of decellularized extracellular matrix

The immunogenicity of the decellularized extracellular matrix (dECM) remains a complex issue, and no single factor can predict whether a dECM scaffold is non-immunogenic with absolute certainty. These factors should be considered when developing and testing dECM scaffolds for clinical applications, because they can affect immunogenicity and transplant failure. The decellularized extracellular matrix (ECM) that has been decellularized (dECM) may contain a variety of immunogens, such as antigenic motifs and protein fragments from the ECM that can interact with host cells and trigger an immune response. The immunogens kappa-elastin, thrombospondin, BM-40, arresten, canstatin, tumstatin, and metastasis are examples of those present in the decellularized ECM. These immunogens function as matrikines that alter the plasticity of healthy monocytes and induce specific immunological reactions. A1b1 integrin, laminin, aggrecan, versican, collagen types I and IV, and hyaluronan are examples of ECM proteins containing hidden antigenic motifs that may support secondary immunity, B-cell development, antibody generation, and chemokine receptor-mediated immune responses [65].

Decellularization can change the structure of the ECM and make it more immunogenic by altering its chemical composition. For example, harsh decellularization techniques involving detergents or solvents may denature ECM proteins and expose covert epitopes that elicit immune responses. Tissue decellularization has been utilized in tissue engineering and regenerative medicine to remove cellular components from tissues, while maintaining the ECM structure. Non-ionic, ionic, and zwitterionic detergents are the three main types used. These detergents have unique processes that damage cell membranes and remove biological components, leaving tissues devoid of cells. Non-ionic detergents, such as Triton X-100, effectively preserve the ECM by preventing DNA-protein interactions and benefit from moderate decellularization, which preserves tissue architecture. However, they may not entirely remove the cellular components, which could result in immunogenicity [66]. Ionic detergents can be used to successfully lyse cell membranes and extract DNA from proteins. One such compound is sodium dodecyl sulfate (SDS). They may require additional procedures for ECM preservation, because they can harm ECM proteins. However, they offer comprehensive decellularization [67]. Zwitterionic detergents such as 3-[(3-Cholamidopropyl) dimethylammonio]-1-propanesulfonate (CHAPS). They offer a compromise between efficiency and ECM preservation by combining the characteristics of both non-ionic and ionic detergents.

The immunogenicity of the dECM may also be influenced by the presence of residual cells or cell debris. Decellularisation can result in cell death. However, DNA and other cellular components are still present in the ECM and are recognized by the immune system [68].

Specific requirements must be met for a tissue to be entirely decellularized, including DNA and GAG (glycosaminoglycan) content. These standards guarantee the elimination of cellular components while protecting the extracellular matrix (ECM) for prospective tissue engineering and regenerative applications. Ideally, the DNA concentration in the decellularized tissue should be below a specified level. The remaining fragments from properly decellularized tissue often have DNA concentrations below 50 ng/mg and are less than 200 bp in length. The residual DNA content required for clinical application is 50 ng/mg of tissue [69]. GAGs in the extracellular matrix are crucial elements of the ECM. Although there is no set standard for the GAG content during decellularization, effective decellularization techniques attempt to maintain a sizable amount of the original GAG content to preserve the structural and functional features of the tissue [70]. The retention of the GAG content during the decellularization process has been shown to be one of the most effective uses of TRITON-X among the various decellularization agents studied [71].

Neoantigens are derived from somatic mutations in cancer cells, which generate new antigens that can induce an antigen-specific T cell immune response for cancer immunotherapy. Decellularization leaves antigens within the ECM, and damage-associated molecular patterns (DAMP) induce M1 macrophage polarization. C3a, C3b, and C5a recruit immune cells and induce T helper cell polarization. T cell activation leads to B cell maturation, antibody production, and complement activation. Neoantigens have high immunogenic potential and can elicit an immune response even in individuals who have never been exposed to the organ [72].

Different natural sources, including rat and human breast adipose tissue, have been used to create self-gelling dECM hydrogels that support tumor organoid growth. Engineered dECMs have been explored for their potential in providing tailored mechanical and biochemical cues for organoid growth [73]. Matrigel, derived from the Engelbreth-Holm-Swarm (EHS) mouse sarcoma, is a widely recognized ECM protein-based hydrogel used as a “golden standard” for organoid expansion. Engineered matrices with a defined composition offer control over cell-matrix interactions but may lack some natural cues. Hydrogel-based matrices exhibit tunable physical properties. Achieving tissue-specific biochemical cues remains a challenge. Matrigel is a widely used universal matrix for various types of organoids including breast organoids. The undefined composition of matrigel can introduce batch-to-batch variability [53]. These limitations include potential immunogenicity, incomplete removal of cellular components, batch variability, and challenges in mimicking complex native ECM structures [74].

### Role of collagen and fibronectin as ECM components and integrin-mediated downstream signaling

In this section, we discuss various biological processes through which ECM stiffness alters cell behavior, including uncontrolled proliferation, metastasis, angiogenesis, and resistance. The ECM is a major regulator of cell behavior. The composition and organization of mammary gland ECM are modified and altered as BC (Breast cancer) progresses. In a soft matrix, tumor cells proliferate more slowly, whereas the stiffness of the matrix promotes the growth of cancer cells via several signaling pathways [75–77]. The evolutionarily conserved serine/threonine kinase signaling cascade, known as the Hippo pathway, was first discovered in the fruit fly, *Drosophila melanogaster*. The Hippo pathway and Salvador-Warts Hippo (SWH) are important pathways involved in cancer cell proliferation [78]. Mammalian Ste20-like kinase 1/2 (MST1/2), large tumor suppressor 1/2 (LATS1/2), and yes-associated transcriptional regulator/tafazzin (YAP/TAZ) are the three molecules that constitute this pathway. Yes-associated protein 1 (YAP) and transcriptional coactivator with PDZ-binding domain (TAZ) are two orthologs of *Drosophila Yorkie*, whose activity is negatively regulated by the Hippo pathway [79]. When matrix stiffness develops, collagen binds to integrin (cell surface receptors) because of increased integrin-linked kinase (ILK)-integrin signaling, which increases the phosphorylation of myosin phosphatase target subunit 1 and suppresses its activity, leading to the suppression of a signaling cascade comprising NF2/Merlin, MST1/2, and LATS1/2 [80]. Focal adhesion signaling molecules, such as FAK, Src, paxillin, Rac, Rho, and Ras, are also recruited by collagen-induced integrin clustering, causing cancer cell proliferation [81, 82]. Protein kinase A (PKA) and p21-activated kinase (PAK) specifically inactivated NF2/merlin by phosphorylating the S518 residue in the tail domain, whereas myosin phosphatase (MYPT1-PP1) activated merlin by dephosphorylating the S518 residue. Merlin activates MST kinases via the phosphorylation of MST1 at Thr183 and MST2 at Thr180 in the MST dimer activation loop. MST kinases have a unique coiled-coil structure at their carboxyl-terminus known as the SARAH domain. MST1/MST2 homo- and heterodimerization are mediated by the SARAH domain [58]. The MST1/MST2 heterodimers form a complex with the SARAH domain-containing protein Salvador 1 (SAV1). MST1/MST2 kinases phosphorylate and activate the LATS1 and LATS2 kinases at Thr1079 and Thr1041, respectively. MST1/MST2 kinases phosphorylate MOB1A (Monopolar Spindle one, binder protein) and MOB1B at Thr35 and Thr12, respectively, which facilitates their interaction with LATS1 and LATS2 [83, 84]. Activated LATS1 and LATS2 This phosphorylates YAP and TAZ and leads to their binding to 14–3-3 proteins, resulting in the cytoplasmic sequestration of YAP/TAZ or ubiquitin-mediated protein degradation [85–87]. When LATS1/LATS2 kinases are not activated, YAP/TAZ are not phosphorylated and translocate to the nucleus. Although YAP/TAZ lacks a DNA-binding domain, it interacts with the TEAD transcription factor family (TEAD1–4) to mediate the expression of target genes such as connective tissue growth factor (CTGF) and cysteine-rich angiogenic inducer 61 (CYR61) to support cell growth, proliferation, migration, and survival [88].

**Fig. 2:**
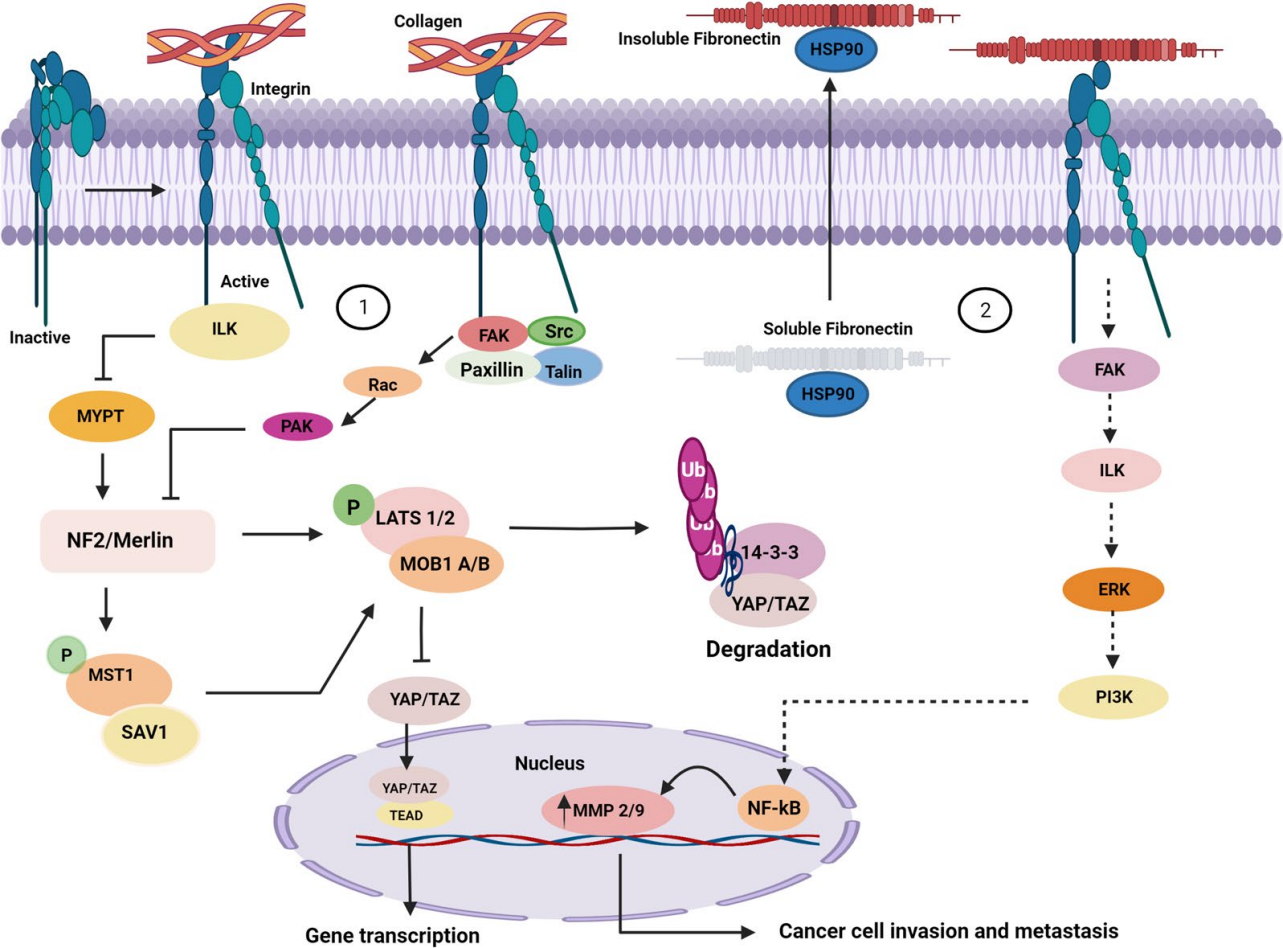
Schematic representation of the role of collagen and Fibronectin as ECM components and integrin-mediated downstream signaling 1) ILK prevents the activity of myosin phosphatase target subunit 1 (MYPT), which results in the inhibition of the Hippo signaling pathway. This inhibition ultimately triggers gene transcription and cell proliferation through the YAP/TAZ transcriptional co-activators. 2) HSP 90 functions as a chaperone aiding in the stabilization of fibronectin. This support leads to the conversion of soluble fibronectin into an insoluble form. The insoluble fibronectin, in turn, plays a role in initiating cell invasion and metastasis by activating a series of pathways, including FAK, ILK, ERK, PI3K, and NF-κB cascades

ECM glycoproteins are present in small quantities and perform a wide range of functions. Fibronectin is secreted by the hepatocytes into the circulatory system as a soluble dimer [89]. HSP 90 functions as a chaperone and aids the stabilization of fibronectin. This supports the conversion of soluble fibronectin into an insoluble form [90]. This phenomenon has also been reported in patients with breast cancer. Elevated levels of fibronectin induce the invasion and metastasis of breast cancer via the activation of a series of pathways, including the FAK, ILK, ERK, PI3K, and NF-κB cascades [91]. (Fig. 2).

### Hyaluronic acid-mediated regulation of cell migration, invasion, differentiation and metastasis

**Fig. 3:**
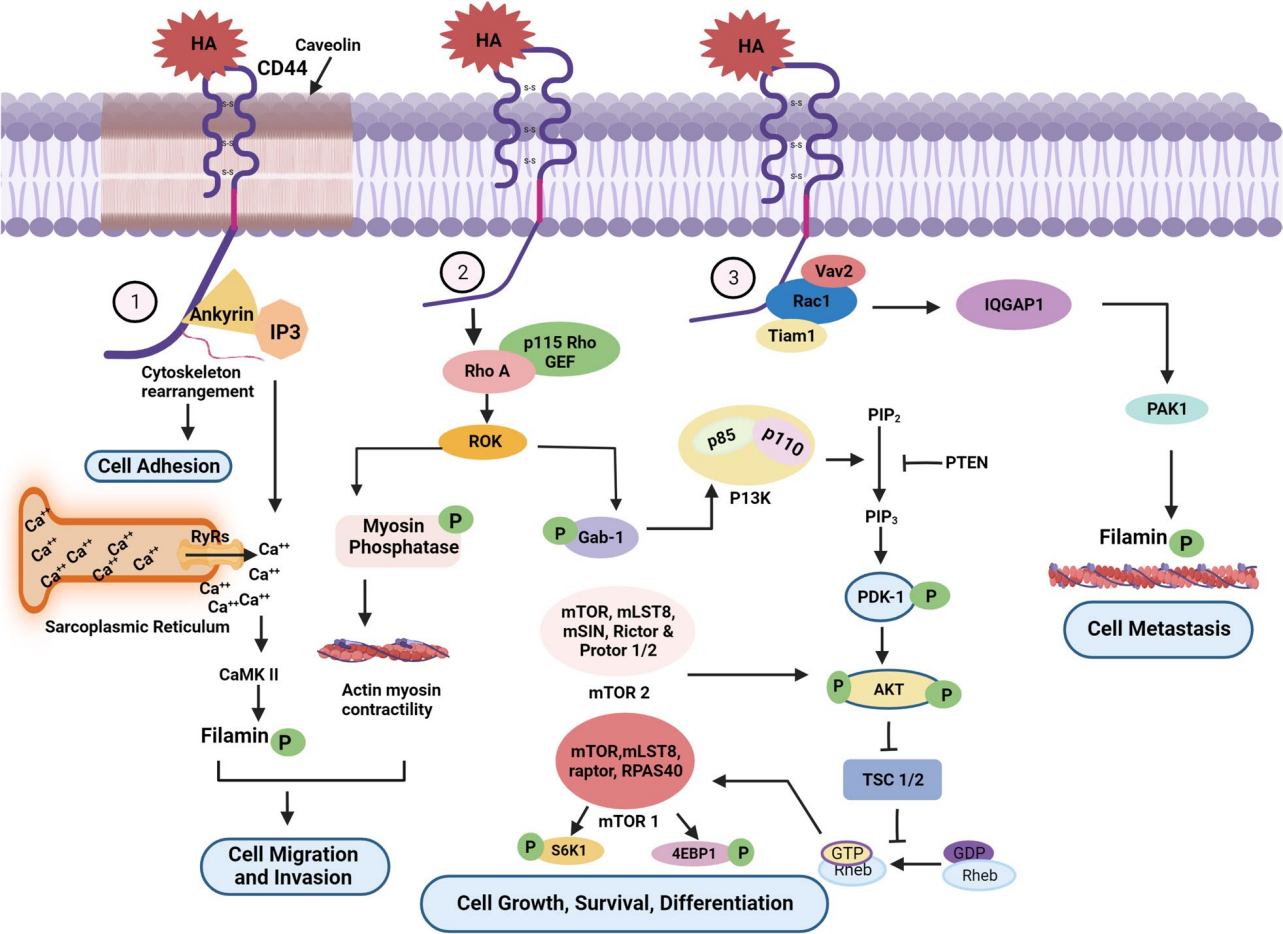
Schematic representation of Hyaluronic acid-mediated regulation of cell migration, invasion, differentiation and metastasis. 1) The interaction between HA and CD44 triggers the activation of ankyrin, leading to cytoskeleton rearrangement and facilitating cell adhesion. Ankyrin also plays a role in the release of calcium, which binds to the calmodulin II receptor. This binding event subsequently leads to the phosphorylation of filamin, promoting processes such as cell migration and invasion. 2) HA by binding with CD44 activates RhoA, which in turn, phosphorylates ROK (Rho-associated protein kinase) and initiates chain of events that contribute to cell growth, survival, and differentiation. These effects are achieved through the activation of myosin phosphatase, elevation of cellular acidity (lower pH), and enhancement of the PI3-AKT signaling pathway 3) HA and CD44 interaction induced the activation of Rac1, which subsequently promotes cell metastasis

In this section, we describe how hyaluronic acid (HA), an ECM component, induces cell adhesion, migration, invasion, differentiation, and metastasis. HA, a significant constituent of ECM, is a large molecule comprising repeating units of N-acetylglucosamine and glucuronic acid. Within the ECM, HA serves as a crucial “reservoir” for water, buffering ion exchange and osmotic balance Fig 3.

#### Interaction of HA-CD44 with cytoskeletal protein ankyrin

Ankyrins are a class of adapter proteins that connect the submembranous actin/−spectrin cytoskeleton to integral membrane proteins [92]. Ankyrin has three functional domains: a spectrin-binding domain, variable-sized C-terminal regulatory domain, and conserved N-terminal ankyrin repeat domain (ARD). ARD is composed of 22–24 tandem repeats of 33 amino acids with a consensus sequence, G–TPLH, AA, GH, V/A, LL, GA, and ND. A number of crucial HA-mediated processes, including cell adhesion, proliferation, migration, and cytoskeleton activation, are triggered by the CD44-ankyrin interaction [93]. Lateral compartmentalization of molecules at the cell surface is carried out by lipid rafts and plasma membrane microdomains rich in sphingolipids and cholesterol. Electron microscopy revealed caveolae, plasma membrane invaginations (lipid rafts) 60–80 nm in diameter. Smooth muscle, fibroblasts, endothelial cells, and adipocytes are only a few examples of diverse tissues and cell types in which caveolae are expressed. Endocytosis, transcytosis, calcium signaling, and modulation of numerous signaling processes are functions of caveolae. Caveolae contain caveolin, cholesterol, and sphingolipids, and caveolin has been observed to colocalizes with both CD44 and ankyrin in lipid rafts [94]. Ankyrin interacts with the IP3 receptor to facilitate calcium release from the sarcoplasmic reticulum through the Ryanodine receptors (RyRs) receptor. The liberated calcium then binds to the calmodulin receptor II, leading to filamin phosphorylation. This process enhanced cell migration and invasion [95].

#### Rho a signaling by the interaction of HA-CD44 for cell migration and invasion

The Rho GTPase family of proteins belongs to the Ras superfamily. Rho GTPases are highly conserved in almost all eukaryotes and support a number of cellular functions, such as control of gene expression, development of the cell cycle, cell growth, cell survival, cell invasion, and cell migration [96]. RhoGEFs (guanyl exchange factor) are required for the activation of Rho A. RhoGEFs have two domains: the Dbl homology (DH) domain that binds to Rho GTPases, while the pleckstrin homology (PH) domain supports the catalytic activity of the DH domain. There are 3 Rho A-specific GEFs have been found to control HA-mediated CD44 signaling during tumor cell activation: p115-RhoGEF, leukemia-associated RhoGEF (LARG), and PDZ-RhoGEF [97]. RhoA interacts with downstream effectors, such as Rho-associated coiled-coil containing kinases (ROK/Rho kinase/ROCK). ROK, a serine-threonine kinase, has a molecular weight of 158 kDa, belongs to the AGC family, and consists of various domains such as a Rho-binding domain (RBD), a PH domain, and a catalytic kinase domain located in a coiled-coil region near the N-terminus. ROK also exhibits autoinhibitory activity by binding to its N-terminus. By interfering with the auto-inhibitory action of N- and C-terminal binding, active RhoA binds to and activates the RBD domain of ROK [98–100]. Myosin II regulatory light chain phosphatase (MLCP) activity is inhibited by activated ROK in a phosphorylation-dependent manner. As a result, increased amounts of phosphorylated and active MLC mediate the assembly of actomyosin and cause actin-myosin contractility, cell migration, and invasion [101].

#### ROK-mediated (PI3K)/AKT/mammalian target of rapamycin (mTOR) signaling

The phosphatidylinositol 3-kinase (PI3K)/Akt/mammalian target of rapamycin (mTOR) signaling pathway plays a crucial role in many cellular processes, such as cell growth, survival, and differentiation. (PI3)/Akt is abnormally active in breast cancer and promotes tumor growth and development. Active ROK phosphorylates the adaptor protein, Gab-1. Gab-1 phosphorylation increases PI3K recruitment [102]. PI3K belongs to a group of plasma membrane-associated lipid kinases that consists of three subunits: the p85 regulatory subunit, the p110 catalytic subunit, and the p55 regulatory subunit. When PI3K is activated, it phosphorylates PtdIns(4,5) P2(PIP2) to generate PtdIns(3,4,5) P3(PIP3) [103, 104]. Phosphatase and Tensin Homolog deleted on Chromosome 10 (PTEN) is an enzyme with the ability to dephosphorylate both proteins and lipids. It is encoded on chromosome 10q23. Structural analysis of PTEN has revealed two key domains: a C2 domain that attracts membrane phospholipids and a phosphatase domain featuring the hallmark CX5R pattern common among phosphatases [105–107]. PTEN inhibits PIP 3 by dephosphorylating PIP3, which phosphorylates the conserved serine (S241) in the activation loop of PDK1(3-phosphoinositide-dependent kinase 1) and leading to PDK1. PDK1 consists of two domains: an N-terminal kinase domain and C-terminal phosphoinositide-binding PH domain [108]. AKT, a serine/threonine kinase also known as protein kinase B (PKB), is phosphorylated by PDK1 at Thr308 and by the mechanistic target of rapamycin complex 2 (mTORC2) at Ser473 in the plasma membrane and is activated. mTORC2 is composed of mTOR, Rictor (a rapamycin-insensitive companion of mTOR), mammalian Sty1/Spc1-interacting protein (mSIN), mLST8, Protor1, and Protor2 [109, 110]. AKT decreases during the assembly of TSC1/2 (tuberous sclerosis complex (TSC) 1/2) complex. This inhibits the activation of RHEB, a member of the RAS family. Rheb activates mTORC1 [111]. The mTORC1 complex is composed of mTOR, mLST8, raptor, and PRAS40 and promotes cell growth, survival, and differentiation by phosphorylating S6 kinase 1 (S6K1) and eIF-4E-binding protein 1 (4EBP1), two well-known regulators of protein synthesis [112].

#### HA-CD44-dependent metastasis via activation of the Rac (Ras-related C3 botulinum toxin substrate 1)

The small GTPase Rac1 is involved in various dynamic cell biological processes, including cell motility, invasiveness, epithelial-mesenchymal transition (EMT), proliferation, survival, and cell-cell interactions [113]. Tiam1 (T-cell lymphoma invasion and metastasis 1) and Vav2 are two GEFs specific for Rac1 [114]. Tiam1 belongs to the Dbl family of guanine nucleotide exchange factors (GEF) and functions as a specific activator of the Rho-family GTPase Rac1. Tiam1 comprises several domains, including an N-terminal pleckstrin homology coiled-coiled extension, a C-terminal pleckstrin homology domain, and catalytic Dbl homology [115]. Vav2 belongs to the Vav family of oncoproteins that act as GEF for Rac1. VAv2 comprises various domains including Pleckstrin Homology (PH), acidic (Ac), Catalytic Dbl Homology (DH), calponin homology (CH), Zinc Finger (ZF), Src Homology 2 (SH2), and Two Src Homology 3 (SH3) domains [116, 117]. HA promotes the interaction between CD44 and several Rac1-specific guanine nucleotide exchange factors (such as Tiam1and Vav2), which upregulate Rac1. Active Rac1 responds quickly to tumor microenvironment (TME) alterations. Rac1 signaling activates IQGAP1, P21-Activated Kinase 1 (PAK1), and filamin in invasive lymphoma and breast carcinoma cells, resulting in filamin cytoskeleton activation and metastasis [118].

## Multicellular heterotypic breast cancer organoid

The Co-Culture System approach involves pre-culturing various cell types separately and then combining them to allow their self-assembly into spheroids [119, 120]. Mammary epithelial cells can be broadly divided into luminal and basal cells based on their location within the bilayer breast epithelium. To develop organoids that closely resemble the in vivo breast microenvironment, these cells were isolated from breast tissue samples and grown in a three-dimensional culture system. The efficiency of this procedure has recently increased, allowing for the long-term culture of breast cancer (BC) organoids and preservation of several lineages within the breast epithelium, including progenitor cells [121].

Researchers have used genetic manipulation methods in breast organoids to study the biology of breast cancer and drug responses. Oncogenic transformation in various breast cancer subtypes and specific genetic alterations or mutations have been introduced into organoids to mimic tumor characteristics. The progesterone receptor (PR) regulates the expression of various genes involved in cell adhesion, immune response, and survival, such as receptor activators of the NFκB ligand and calcitonin [122]. Specific genetic alterations have also been associated with different histological tumor types, such as inactivation of E-cadherin in lobular breast cancer and HER2 gene amplification in poorly differentiated ductal cancer [123]. Furthermore, germline mutations in BRCA1 and BRCA2 have been linked to genetic predisposition to breast cancer [124]. However, there are concerns and limitations associated with xenotransplantation, including the risk of contamination, and the need for further research and validation before considering clinical applications [125].

Humanized cancer models in rodents involve a combination of mouse models with xenografted or spontaneous human cancer cells, along with the human immune system (HIS) mice. These models have become more sophisticated and robust, allowing for in vivo exploration of human cancer immunology and immunotherapy [126]. The laboratory mouse is the most common animal model used in cancer research because of its genetic variability, physiological similarities with humans, and ability to generate humanized mouse models by incorporating the human immune system with human tumor xenografts [127]. Breast cancer organoids have been xenotransplanted into immunocompromised mice to examine therapeutic interventions, such as non-obese diabetic severe combined immunodeficiency or NOD-scid Mice, which are highly immunodeficient and suitable for the transplantation of human tissues because they lack functional B and T cells. A more sophisticated immunodeficiency model is provided by NOD-SCID IL2Rγnull (NSG) mice, which are deficient in B and T cells and functional NK and IL2R signaling. NSG mice have gained popularity as a popular choice for xenotransplantation studies because of their improved engraftment efficiency. Another strain of mice with multiple immunodeficiencies, including a problem with IL2R signaling, are NOG (NOD/Shi-scid/IL2Rγnull) mice, which are suitable for engrafting human tissues. Similar to NSG mice, NSI (NOD/Shi-scid IL2Rγnull) mice lack the IL2R chain, which enhances their capacity to engraft human tissue [128, 129]. Breast xenotransplantation models have advantages and disadvantages. The breast xenotransplantation model allows for the study of human breast cancer in an animal model and can provide insights into the self-renewal capacity and differentiation potential of distinct cell populations or individual cells in the mammary gland. The disadvantages of xenotransplantation models may not fully replicate the complexity of the human tumor microenvironment. The theoretical hazard of causing new human infections through the intermingling of tissues from different species has been a concern in the field of xenotransplantation [130].

Heterotypic organoids are created by cultivating pluripotent or multipotent stem cells in a three-dimensional (3D) matrix under conditions that encourage self-organization and the presence of various cell types. Organoids are ex vivo multicellular fragments produced by cultivating pluripotent or multipotent stem cells in a 3D matrix under conditions that promote self-organization. These conditions are established experimentally, and frequently use information regarding the signals involved in organ development or regeneration [131–133].

Normal fibroblasts are mesenchymal cells responsible for maintaining tissue homeostasis, whereas cancer-associated fibroblasts (CAFs) are fibroblasts that have been chronically misregulated in epithelial cancers. CAFs are a dominant and heterogeneous cell type within the tumor microenvironment (TME) and play a pivotal role in controlling cancer cell invasion, metastasis, immune evasion, angiogenesis, and chemotherapy resistance [134]. Unlike normal fibroblasts, CAFs have tumor-promoting functions and can influence tumor progression, invasion, and response to therapy. CAFs communicate with cancer cells and other cells in the TME through various mechanisms, including metabolite exchange, paracrine signaling, desmoplasia, and acidosis [134, 135]. CAFs have been found to promote cell survival during detachment, block anoikis, and facilitate luminal filling in three-dimensional cell culture [136]. CAFs also secrete insulin-like growth factor-binding proteins (IGFBPs) that stabilize the anti-apoptotic protein Mcl-1, contributing to anoikis inhibition [137]. Additionally, CAFs promote epithelial-mesenchymal transition (EMT) by secreting collagen triple helix repeat containing-1 (CTHRC1), which activates the Wnt/β-catenin signaling pathway [138, 139]. Human adipose tissue-derived stem cells (hASCs) have been identified as a potential source of CAFs because they can differentiate into a CAF-like myofibroblastic phenotype when exposed to conditioned medium from breast cancer cell lines [140]. Fibroblasts were added to the 3D tissue models to replicate the stromal environment found in the breast tissue and produce a more accurate representation of the breast cancer model. Fibroblasts play a crucial role in maintaining scaffold-free conditions by promoting cell migration and proliferation within a collagen matrix [141]. Fibroblasts also shed microvesicles from their plasma membranes, which then spread throughout the matrix. The presence of fibroblasts provides favorable conditions for simulating collagen processing in vitro and for understanding the mechanisms controlling cell uptake and intracellular degradation [142]. Additionally, fibroblasts encapsulated in a collagen gel show enhanced extracellular matrix (ECM) production, including collagen type I and elastin expression. This suggests that fibroblasts contribute to the maintenance of scaffold-free conditions by actively participating in ECM synthesis and remodeling. Fibroblasts influence the behavior of immune and endothelial cells within the tumor microenvironment. They secrete fibroblast growth factor (FGF), which attracts immune cells to the tumor site and promotes their activation and differentiation. By releasing pro-angiogenic factors that encourage endothelial cell migration and proliferation, fibroblasts also help in the development of new blood vessels that supply the tumor with nutrients.

Endothelial cells play a crucial role in tumor angiogenesis, which involves the development of new blood vessels that supply nutrients and oxygen to the developing tumor. These endothelial cells interact with cancer cells and other stromal cells in the tumor microenvironment to promote vascularization and tumor development. Endothelial cells are essential players in tumor angiogenesis, and their interactions with fibroblasts and immune cells can affect their behavior. The formation of blood vessels and development of tumors can be aided by activated fibroblasts, which can increase the production of proangiogenic factors (PAF) in endothelial cells. Vascular endothelial growth factor (VEGF), which affects endothelial cell behavior by regulating permeability and functionality within the tumor microenvironment, can also be secreted by immune cells [143]. Sustained stress-activated myofibroblasts have an altered secretory phenotype, producing factors, such as TGF-β and VEGF, to promote proliferation and recruit other cells. Macrophages and fibroblasts have physiological functions in tissue homeostasis, immune response, angiogenesis, and wound healing.

Cancer stem cells (CSCs) have been shown to play a critical role in breast cancer initiation, progression, metastasis, and drug resistance. These cells possess long-term proliferative potential and the ability to regenerate phenotypically heterogeneous cell types. CSCs in breast cancer often exhibit attributes of cells that have undergone an epithelial-mesenchymal transition (EMT) [144]. Breast cancer stem cells (BCSCs) are driven by the persistent activation of developmental pathways such as Notch, Wnt, Hippo, and Hedgehog [145]. This trilayer breast organoid serves as a reliable model for studying breast cancer and provides valuable insights into this disease. These organoids enable researchers to better understand the molecular characteristics of breast cancer, which can help assess the therapeutic response. Additionally, trilayer breast organoids have the potential to identify new druggable targets for targeted therapy [146]. Their inclusion in 3D breast cancer models is crucial for a better understanding of tumor angiogenesis and vascular interactions [147].

Various cell types within the organoid, reflecting the cellular variety in the associated organ, are heterotypic aspects of multicellular organoid culture, essential for simulating the interactions and crosstalk between various cell populations in the organ, which supports physiological processes, can be disturbed in diseases such as cancer, and contributes to their maintenance [148].

Scaffold-free breast organoids display characteristics resembling those of normal human breast acini, including a hollow lumen and secondary acini, and express mammary gland-specific progenitor markers [149]. Scaffold-free organoids also have high consistency and reproducibility, as well as the ability to measure cellular collagen I production without noise from exogenous collagen, and can be subjected to various stimuli from the microenvironment and exogenous treatments with precise timing without concern for matrix binding [150]. Additionally, scaffold-free breast organoids can be generated from primary mammary carcinomas, retaining the high-grade spindle cell morphology of the primary tumors.

Breast tumor-derived fibroblasts secrete extracellular matrix (ECM) components that induce morphogenesis and growth of breast epithelial cells. Adipose progenitor cells have been shown to assemble the fibronectin (Fn) matrix in response to soluble factors secreted by breast cancer cells, leading to increased stiffness of the tumor stroma [151]. ECM proteins upregulated in breast tumor tissue were found to have cell line-specific effects on cell migration and invasion, with cell adhesion, elongation, and irregularity being key determinants [152]. Multiple cell types, such as mammary epithelial, tumor, and stromal cells, all of which contribute to the synthesis and remodeling of ECM, are involved in the development of breast organoids. In turn, the ECM maintains organoid structure and functionality by creating a microenvironment that resembles that of both normal breast tissue and tumors. The primary cell types involved in developing breast organoids are mammary epithelial cells, which help produce ECM constituents, including collagen, laminins, fibronectin, and proteoglycans. The ductal and lobular structures found in the mammary gland are maintained by mammary epithelial cells, which also develop epithelial compartments in the organoids.

One study focused on spheroid cell culture methodological factors to improve reproducibility and physiological significance when investigating the metabolic effects of drug treatment in breast epithelial cells. Spheroids were formed by co-culturing MCF10A breast epithelial cells and MDA-MB-231 breast cancer cells in standardized and enriched media (DMEM or RPMI). Spheroid analysis was used to assess metabolic behavior and integrity using Spheroid-Sizer software, confocal microscopy, and western blotting [153]. Extracellular matrix-stromal cell interactions contribute to the neoplastic phenotype of breast epithelial cells. A previous review examined the role of the extracellular matrix and stromal cells in influencing the neoplastic phenotype of epithelial cells during the development of breast cancer. In breast cancer, epithelial-mesenchymal interactions involve stromal microenvironmental factors that influence epithelial growth, hormonal responses, morphogenesis, and plasticity [154]. 3D Cell Structures created a vascular endothelial-breast epithelial cell coculture model. In a study, a 3D model of vascular endothelial-breast epithelial cell interactions was developed, focusing on cell-cell interactions between endothelial and breast epithelial cells. Breast epithelial cells migrated out of their spheroids and along HUVEC networks, which appeared to be partly mediated by secreted EGF and cell-cell contact [155]. Another study comprehensively investigated altered lipid metabolism in breast cancer, examined changes in lipid composition, identified critical regulators, and analyzed their impact on cancer progression. This study revealed novel lipidomic changes in EMT-induced breast cancer and emphasized the importance of ELOVL2 in cancer progression. Cancer-associated fibroblast CAFs, a type of mesenchymal cell found in the tumor stroma, have been shown to require proline synthesis by PYCR1 to deposit a pro-tumorigenic ECM. CAF subpopulations that produce collagen-rich ECM, such as myofibroblast-like CAFs (myCAFs), contribute to tumor progression and metastasis [156]. Tumor cells from patient samples are also a part of the organoid culture in the case of breast cancer organoids. As they still possess the capacity to produce ECM elements resembling those found in the tumor microenvironment, tumor cells contribute to ECM synthesis and remodeling within organoids [157].

Tumor cell secretion of vascular endothelial growth factor (VEGF) in response to hypoxia stimulates endothelial cell proliferation and angiogenesis within the tumor microenvironment [158]. The formation of capillary-like structures during the assembly and growth of tumor cell-endothelial cell (TC:EC) spheroids suggests the formation of a network of blood vessels within these models. This formation is critical for the nutrient supply to growing tumor cells and indicates spatial invasiveness within the ECM. These spheroid shapes and surface textures can provide information regarding the invasive potential of cells within the ECM. These findings emphasize the importance of understanding the dynamic interactions between tumors and endothelial cells in the context of 3D models [159].

Breast cancer is a multifactorial disease that includes many separate entities with markedly different biological characteristics and clinical manifestations. Based on the expression of human epidermal growth factor receptor 2 (HER2) and hormone receptors (HRs) (progesterone and estrogen) breast cancer is categorized into four subtypes: Luminal A, Luminal B, HER2 enriched, and Triple-negative breast cancer (TNBC) [160]. Out of all breast cancers, 50 to 60% are known to be luminal A (LABC; ER/PR+, HER2-, and low expression of Ki-67). This subtype has a great prognosis with limited invasiveness, with a relapse rate that is 27.8% lower than other subtypes [161]. Luminal B is further classified into two types i.e. Luminal B like HER2- (ER+ but ER and PR expression lower than in luminal A-like; HER2-; high Ki67 index) and Luminal B like HER 2+ (ER+ but lower ER and PR expression than luminal A-like; HER2+; high Ki67 index) [162]. About 15–20% of breast cancers are HER2+, which is defined as having evidence of HER2 protein overexpression and determined by immunohistochemistry status (IHC3+), fluorescence in-situ hybridization (FISH) measurement of a copy number of six or more for the HER2 gene, or a HER2/CEP17 ratio of 2·0 or higher. In 2013, the American Society of Clinical Oncology/College of American Pathologists (ASCO/CAP) revised their criteria, reintroducing a cutoff value of 2·0 or above for the HER2/CEP17 ratio and full staining of more than 10% of the cells [163]. TNBC is characterized by the absence of progesterone receptor (PR) and estrogen receptor (ER) expression, as well as the lack of HER2 overexpression and/or gene amplification. According to the 2010 ASCO/CAP recommendation, invasive breast tumors should be classified as ER-positive if their immunohistochemical ER expression is ≥1% [164]. While the therapeutic relevance of several genetic subtypes of breast cancer has been extensively acknowledged, the importance of tumor extracellular matrix heterogeneity has been mainly overlooked [165]. The significance of tissue-specific ECM and tissue-mimicking biomaterials in tissue/organ regeneration has been emphasized by advances in tissue engineering. Tumor-derived extracellular matrix (ECM) may be more effective than tissue engineering at simulating the intricate physiology of the natural microenvironment [166]. Tan et al. 2023 in their research compared the composition, organization, and intended application of ECM obtained from two genetic subtypes of breast cancer: TNBC (very aggressive, ERα-)-derived ECM (TN-ECM) and luminal-A breast cancer (less aggressive, ERα+)-derived ECM (LA-ECM). Through comparison, they discovered that Tumor-derived ECM displayed altered architecture and increased levels of pro-collagen I, fibronectin, and laminin compared to normal breast tissue-derived ECMs (B-ECM). They also explored that HER2+ tumor subtypes have been related to higher collagen deposition levels. In TNBC and HER2+ breast cancers, fibronectin was substantially expressed in both the primary and metastatic tumors [167]. These results highlight the significance of tissue-mimicking microenvironments in drug testing by potentially clarifying the distinct microenvironments linked to native tumor matrices. *Rafaeva, Maria* et al. Fibroblast-derived matrix (FDM) model. By employing this model, they demonstrate that, in contrast to FDMs originating from non-malignant tissue (normal) fibroblasts, cancer cells exhibit enhanced proliferation on cancer-associated FDMs. At the primary tumor site, they evaluate changes in ECM characteristics from normal to cancer-associated stroma [168]. There are presently very few temporally resolved proteomic studies available, that more accurately reflect deposited extracellular matrix during the course of disease progression. Their FDM proteomics approach can be used to bridge this gap by investigating the ECM deposition by the CAF subtypes.

**Fig. 4:**
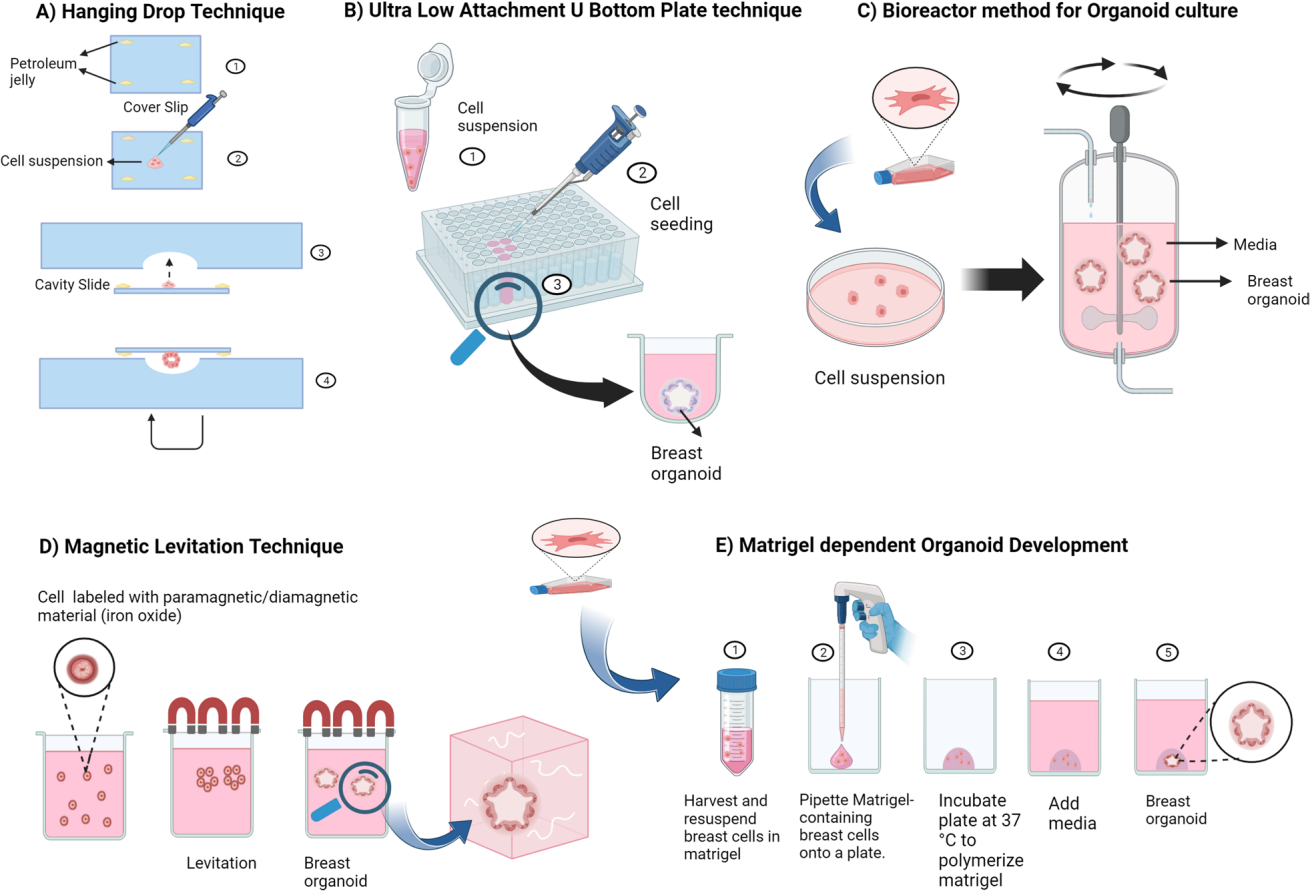
Schematic representation of Variable organoid development techniques. **A** The hanging drop technique, allows organoids to self-assemble into 3D structures by dropping small drops of cell-containing liquid upside down on a culture surface. **B** The ultra-low attachment U-bottom technique is used to produce organoids by placing cells in U-shaped wells with non-sticky surfaces and stimulating them to develop 3D structures without attaching to the bottom. **C** The bioreactor method for organoid culture involves placing cells in a controlled environment that simulates the conditions of the body, allowing them to develop into more realistic and functioning 3D structures. **D** Magnetic levitation for organoid development involves suspending and arranging cells by virtue of magnetic force. **E** In Matrigel-dependent organoid development, cells are implanted in a gel-like substance called Matrigel, which acts as a scaffold to stimulate the production of 3D organoids by mimicking the natural cellular environment

**Fig. 5:**
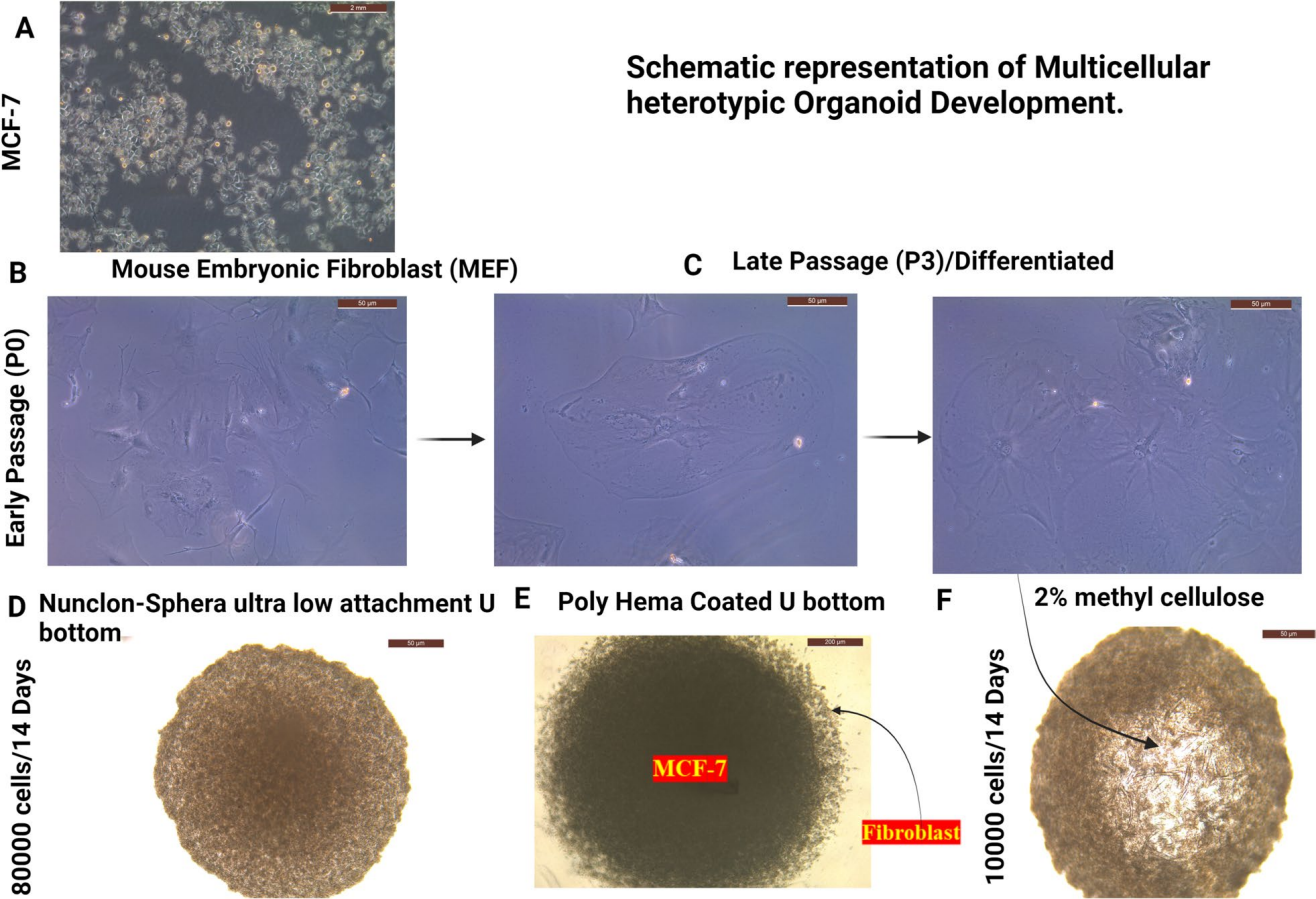
Schematic representation of involved variable process for development of multicellular heterotypic 3D breast cancer Organoid. **A** MCF-7 cells cultured on 2D monolayer. **B** Early and (**C**) Late stage of primary mouse embryonic fibroblast. Variable organoid developed by help of Nunclon Sphera ultra-low attachment (**D**), Poly-HEMA coated (**E**) and 2% methyl cellulose mediated (F) with a MCF-7 and Fibroblast cell population (80,000 cells and 10:1 cell ratio, 14 days)

**Fig. 6:**
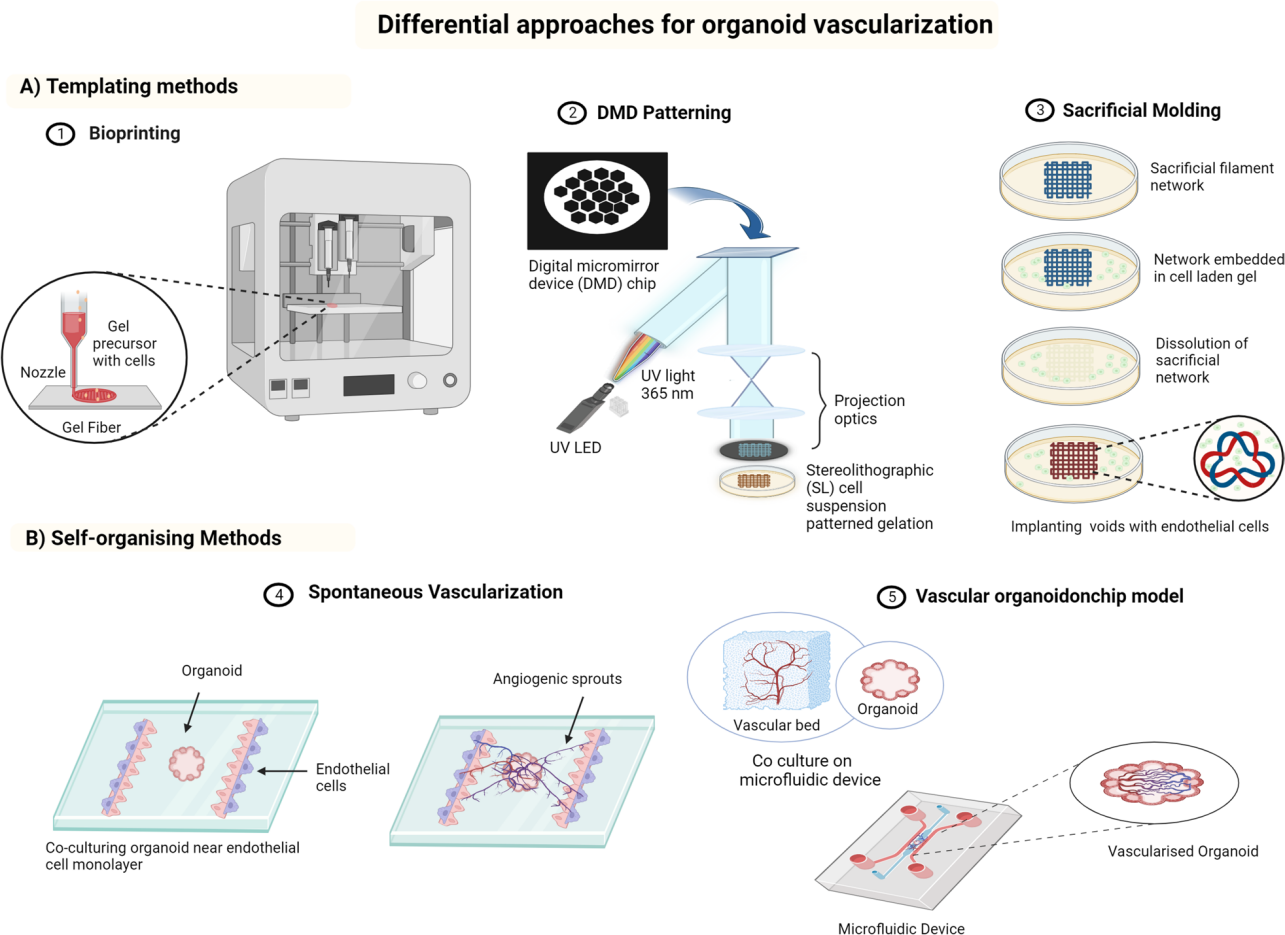
Schematic representation of differential approaches for organoid vascularization. **A** Templating techniques guide the development of vascular networks in organoids 1) 3D bioprinting utilizes precise 3D Biofabrication to stimulate vascularization within organoids. 2) DMD (Digital Micromirror Device) patterning employs micromirrors for precise modulation light and produces vascularized networks within organoids 3) Using the sacrificial network templating method, temporary structures are developed to direct vascular development in organoids. **B** Through intrinsic cellular connections, the self-organizing technique promotes spontaneous vascularization inside organoids. 4) Endothelial cell co-culture in compartmentalized microfluidic systems that facilitate in vitro organoid vascularization 5) Use of controlled fluid dynamics in organoids on microfluidic devices to encourage the development of blood vessels

*Campaner* et al. developed patient-derived organoids (PDOs) derived from various subtypes of breast cancer (luminal A, luminal B, HER2-enriched, and TNBC)*.* Through the application of Masson’s trichrome histochemical staining, which enables the assessment of extracellular matrix deposition, they observed that thesss tumor tissue was characterized by desmoplastic stroma that was enhanced by an excessively fibrous collagen matrix [61]. PDOs can serve as in vitro platforms for testing combination treatments meant to overcome drug resistance as well as for evaluating the sensitivity of cancer cells to conventional therapy. *Charles* et al. in their research revealed that, regardless of patient age or race, collagen 1 (COL 1) expression elevated considerably in most ER+/PR+ breast cancer subtypes. To objectively prove a correlation between fibrillar COL expression and receptor status. RNA sequencing from the SCANB and TCGA data sets was utilized to assess the expression of COL1A1 and COL1A2 in HER2+ and ER+/PR+ cancers. ER−/PR-cancers exhibited significantly (*p* < 0.0001) lower expression of COL1A1 and COL1A2 than ER+ tumors. No correlation was found between the expression of COL1A1 and COL1A2 and HER2 status [169]. The disadvantage of this study is that they used a single 3-day time point for endpoint analysis. Cells had time to adjust to the new culture conditions at this point. Although induced protein changes were not identified, more time points could be needed to observe the translational effects of matrix adherence. Additionally, the use of monoculture, which is not representative of the heterogeneous cell population noticed in vivo, was a drawback of this work. **(**Figs. 4, 5, 6**) (**Table 2**)**

**Table 2:** Significant mammary organoid study with associated protocol details

Serial no.	Study	Protocol Details	Ref.
1	Organoid cultures from normal and cancer-prone human breast tissues	This investigation looks at the early oncogenic transformation of various breast cancer subtypes using long-term culture of BC organoids. Human mammary epithelial cells (HMECs) from different lineages are cultured according to the specified protocol for an endless period.	[2]
2	Long-term culture, genetic manipulation, and xenotransplantation of human normal and breast cancer organoids	The protocol provides information on the long-term culture of normal and breast cancer organoids, genetic modification, and xenotransplantation for investigating tumorigenesis using patient-relevant cancer drivers and mutations.	[128]
3	Current Status of Breast Organoid Models	The study discusses breast organoids as 3D simulations of the in vivo breast microenvironment and investigates the factors influencing breast cancer development.	[157]
4	A Mammary Organoid Model to Study Branching Morphogenesis	Organoid models made from primary mammary epithelial cells are used in this study to examine the branching morphogenesis of the mammary gland. It seeks to comprehend the signaling cues responsible for promoting branching morphogenesis.	[170]
5	Establishment and long-term culture of mouse mammary stem cell organoids and breast tumor organoids	The protocol explains how to maintain the self-renewal of mammary stem cells (MaSCs) that form glands and how to model tumorigenesis by introducing cancer drivers and mutations relevant to patients.	[171]
6	Organotypic culture assays for murine and human primary and metastatic-site tumors	The study offers valuable suggestions for long-term time-lapse imaging of epithelial morphogenesis in three-dimensional organotypic cultures. It sheds light on how the ECM microenvironment controls the mammary epithelium’s collective migration and dissemination.	[172]
7	BRCA-deficient mouse mammary tumor organoids to study cancer-drug resistance	The study examines cancer drug resistance using BRCA-deficient mouse mammary tumor organoids. The organoids are used to simulate basal-like breast cancer and analyze the role of BRCA2 and p53 as tumor suppressors in breast cancer.	[173]
8	Generation and functional characterization of murine mammary organoids	The protocol details experimental techniques for developing mice mammary organoid lines isolated from mammary glands or tumors caused by mutations in PI3K pathway components. These organoids can monitor drug responses and guide more effective treatments.	[174]

## Development of vascularized organoids

Microvasculature integration with parenchyma and breast tissue organoid stroma is required to develop vascularized breast organoids. This is necessary for accurately simulating the native tissue environment, enabling physiologically relevant perfusion of the organoids, and supporting cellular dynamics within the tissue model via perivascular niche cells [175, 176]. Vascularization of breast organoids can be performed in various ways. One method to develop a vascular network inside organoids is to coculture organoids with microvessels or endothelial cells. This method enables the integration of the microvasculature with the breast tissue model, allowing for perfusion and nutrient supply to the cells. Microinjection methods or exposing organoids to endothelial cells in a two-dimensional (2D) layer can be used to incorporate microvessels.

Providing structural support and regulating cellular behavior are essential functions of the extracellular matrix. Vascularized breast organoids may use ECM components to promote vascularization and tissue development [177]. Collagen-alginate hydrogels with filamentous architectures have been used to mimic the ECM of breast tumor microenvironments in the context of breast cancer spheroids and organoids. These hydrogels vary in stiffness by varying the crosslinking of alginate molecules, which influences the mechanical properties of the ECM. The filamentous architecture of collagen–alginate hydrogels mimics the ECM structure in a breast tumor environment. It has been used to study the growth of breast tumor spheroids and their response to chemotherapy [178]. Collagen-rich ECM environments have been shown to promote cell growth and behavior, including those of vascular endothelial cells. In tissue cultures, collagen within the ECM can provide cues for angiogenesis and vascularization, which are critical for the development of functional blood vessels within spheroids and organoids.

Once vascularized breast organoids have been developed, it is crucial to maintain a perfusion system that enables constant circulation of nutrients and oxygen within the tissue model. Long-term tissue viability and functionality were preserved by perfusion.

Microfabricated and microfluidic platforms such as microfluidics and microprinting offer promising tools for addressing organoid and spheroid production limitations. These platforms can improve the nutrient delivery and culture conditions, thereby producing more uniform and reproducible organoids and spheroids. This makes it possible to create size-controlled culture areas that enhance vascularization. However, these techniques may be challenging to implement and require specialized equipment [179]. The co-culture of pluripotent stem cells and endothelial cells on 3D substrate matrices has been proposed to produce vascularized organoids. This method enables the creation of functional organoids with a more accurate representation of corresponding tissues. It allows for the study of physiological processes and disease manifestations in a controlled in vitro environment. However, it is difficult to optimize culture conditions and precisely integrate vascular networks [180]. Researchers have utilized spheroid-based engineering to generate the human vasculature in mice. This approach involves creating 3D spheroids of cells that mimic the tissue architecture and subsequently implanting them into a living host. The advantage of this method is its ability to generate a functional vasculature in vivo. However, controlling the precise formation of vascular networks may be challenging, and host factors can influence outcomes.

Vascularized breast cancer organoids can be used as living biobanks to evaluate drugs and to develop individualized treatments. They are helpful tools for researching drug responses and developing specialized therapies because of their capacity to mimic the tumor microenvironment and heterogeneity of individual patients [181]..

Organoid vascularization is necessary to improve biological relevance and to ensure sufficient oxygen and food supply [182]. Organoid vascularization approaches can be classified into two types: in vitro and in vivo approaches. In the in vivo method, nonvascularized organoids are inserted and left to be vascularized by the host’s peripheral vascular system. To ensure that organoid cells have access to sufficient nutrients for survival, this approach depends on timely invasion of the host vasculature into the non-vascularized organoid through angiogenic sprouting. Naturally, organoids would require more time for vascularization when this method is used. The time required for in vivo vascularization may be too long, resulting in necrosis before the development of a functional vascular network. This limitation has led to the use of in vitro vascularization procedures to develop organoids that are pre-vascularized before implantation, which has definite advantages over nonvascularized organoids [183]. In vitro vascularization can be achieved by co-cultivation of vascular cells or tissue engineering. In vitro vascularization techniques can be divided into templating and self-organizing approaches [184, 185]. Templating methods include 3D bioprinting, DMD patterning, and sacrificial molding. On the other hand, self-organizing methods include co-culture of organoids with endothelial cells in a compartmentalized chamber and neo-angiogenesis in a microfluidic device using control fluid dynamics [186].

### In vitro Templating methods for vascularization of organoids

#### 3D bioprinting

Any additive manufacturing technique that uses biological ink to print living tissue constructs for a number of applications, such as regenerative medicine and cellular investigations, is referred to as “bioprinting.” [187] The three primary 3D bioprinting methods are extrusion-based, inkjet, and laser-assisted bioprinting (LaBP) [188, 189]. In 3D bioprinting, bioinks are crucial components that are cross-linked or stabilized during or immediately after bioprinting to produce desired tissue constructs. A bio-ink is a blend of biologically active molecules, biological materials, and cells. Hydrogels, decellularized matrix components, cell aggregates, and microcarriers are the four main types of bioink materials. Gelatin, hyaluronic acid, silk proteins, and elastin are examples of the natural polymers found in bioinks. Synthetic polymers found in bioinks include amphiphilic block copolymers, polyethylene glycol (PEG), and polyphosphazenes [190–192].

Several studies have focused on adding stem and endothelial cells to prints, selecting bioinks based on physical qualities, and choosing printing techniques based on the physical properties of the desired tissue to aid in the effective development of bioprinted tissue and its vascularization. Hydrogels are frequently employed as bioinks because of their capacity to replicate the ECM and offer an environment that is favorable for cell growth and development. They have strong biocompatibility and can be crosslinked to form a solid structure. Although they are not suitable for all applications, they possess mechanical properties. Alginate is a well-linked bio-ink substance made from seaweed. It has excellent biocompatibility and is simple to crosslink to form a stable structure. It may need to be modified to improve cell attachment because it lacks cell-specific adhesion sites. Another commonly used bioink material with high biocompatibility and cell adhesion qualities is gelatin, which is produced from collagen. However, they may only possess modest mechanical stability and strength. Fibrin is a natural bioink that is produced from thrombin and fibrinogen. It can simulate how blood naturally clots, and encourages cell adhesion and differentiation. It may have only low mechanical strength and stability. A bioink sold and made from the basement membrane of EHS mouse sarcoma cells is called Matrigel. ECM proteins and growth factors that promote cell adhesion and differentiation are also present. However, it can vary from batch to batch and is expensive. PEG and PCL are synthetic polymers with adjustable mechanical characteristics that can be functionalized to improve vascularization and cell adhesion. However, they may be unable to replicate the natural ECM environment [193–196]. Because it is simple to polymerize and offers a suitable matrix for cell development, rat-tail collagen is frequently employed in 3D bioprinting investigations [197]. Alginate can form hydrogels when crosslinked with divalent cations. However, it lacks cell adhesion sites; therefore, other polymers, such as PCL and gelatin, are often mixed with alginate to form different structures [198]. For bioprinting, a marine polymer called agarose derived from seaweed was used as the starting material. Although they have adequate mechanical qualities, their capacity to promote cell development is limited [196].

#### DMD (digital micromirror device) patterning

DMD is a highly effective tool for photostimulation applications, such as photoconversion and optogenetic manipulation. This is because of their strong capability to produce innovative illumination patterns with exceptional spatiotemporal precision. DMDs comprise of rectangular arrays of hundreds to millions of small mirrors that may be tilted between ‘on’ and ‘off’ state by around 12 ° each. A multimode fiber (MMF) with a 50-μm core diameter collected the light pattern from the DMD [199, 200]. In DMD patterning, a liquid gel precursor can be repeatedly exposed to projected sequential light patterns to produce desired 3D tissue structures [201].

For vascularized organoids, sacrificial perfusion networks can be created using DMD-based tools. These networks are produced using micro-stereolithographic techniques, in which a network of branching rods made of a water-soluble photopolymer is polymerized using a proprietary DMD-based 3D printing apparatus. Collagen was then applied to the constructed structure and inserted into the porous scaffold. The network was disintegrated to generate a co-culture model system in a NaOH-containing solution, leaving behind a vascularized scaffold that may be seeded with endothelial cells (HUVECs). This method integrates materials and fabrication technologies to attain the required features in intricate co-culture platforms [201]. Vascularized breast organoids can be produced using a Decellularized Macroporous Device (DMD). By decellularizing cancer-associated fibroblasts (CAFs) cultivated on three-dimensional macroporous polymer scaffolds, researchers have created a biochemico- and mechano-mimetic 3D culture platform for primary breast cancer cells. Cell adhesion and vitality were aided by the extracellular matrix from the CAF placed on the polycaprolactone scaffold. Single cells from primary breast tumors grow and self-organize on this scaffold to form tumoroids. The DMD platform makes it possible to accurately recapitulate tumor behavior and medication response, making it a potential ex vivo platform for primary cell culture and creating efficient and individualized chemotherapy regimens [202, 203].

#### Sacrificial Moulding

In this technique, 3D-printed sacrificial molds or sacrificial networks are used for the vascularization of organoids or to design the desired tissue constructs [204]. Various biomaterials have been used to develop the sacrificial networks. These biomaterials exhibit several unique properties based on physical crosslinking principles. For example, gelatin and polyvinyl alcohol (PVA) can be easily removed by submerging them in water, and a concentrated Pluronic F127 solution can gel at temperatures above 10 °C and liquefy at 4 °C, whereas a concentrated gelatin solution gels at temperatures below 30 °C and liquefies at 37 °C [205]. For organoid vascularization, sacrificial networks are cast into an endothelial-cell-laden gel matrix. The sacrificial template was removed under appropriate conditions once the scaffold elements were fully gelled to provide perfusable channels [206].

Sacrificial molding is a technique used to develop vascularized organoids by using sacrificial templates. This technique uses a thermoset composite with sacrificial materials, such as polyethylene glycol (PEG) elastomers and polylactic acid (PLA). A catalyst such as tin (II) oxalate (SnOx), which undergoes thermal depolymerization and vaporization at a particular temperature, is then applied to the sacrificial material. A network of vasculature that is exactly opposite to the sacrificial template is produced by this vaporization process. Desirable features, including thermal regulation, magnetic or electrical modulation, and in-situ chemical species reactions, can be imparted on the composite by adding functional fluids to the microvasculature. This method enables the fabrication of various vascular and porous structures by allowing size and dimensionality customization across multiple applications [207]. Sacrificial molding using a glucose-sensitive self-healing hydrogel can be used to create vascularized organoids. Borax serves as the glucose-sensitive motif in the hydrogel and is composed of reversibly crosslinked poly (ethylene glycol) diacrylate and dithiothreitol. The hydrogel can be quickly removed by submersion in the cell culture medium and is mechanically robust and injectable. In this study, branched tubular channels were created inside a construct using hydrogel as a sacrificial material. Vascular endothelial cells can line the channel wall and migrate into the non-sacrificial hydrogel after implantation in the channel lumen by perfusion. Endothelial cells gradually developed a capillary-like structure, forming a vascular network within the construct. By employing sacrificial molding and a glucose-sensitive hydrogel, this method enables the fabrication of vascularized organoids such as a neurovascular unit [208].

### In vitro self-organization methods for vascularization of organoids

#### Spontaneous vascularization

For spontaneous vascularization of organoids, the organoid was placed in the center of a compartmentalized microfluidic chip with three parallel fluidic channels separated by microposts, permitting cell migration and proliferation between the channels. The organoid was then co-cultured with endothelial cells, which formed a network around and within the organoid, resulting in fully perfusable vasculature [209, 210].

Different materials can be used to induce spontaneous vascularization in breast organoids. Decellularized cancer-associated fibroblasts (CAFs) were cultivated on macroporous polymer scaffolds. Additionally, the creation of the developed matrices can offer biochemical and biophysical characteristics that facilitate vascularization in organoid cultures. These designed matrices can be natural, synthetic, or protein-engineered hydrogels, and can be adjusted and optimized to support the growth and maturation of organoids [211]. To maintain spontaneous vascularization in breast organoids, it is important to incorporate the vasculature into the culture system. Vasculature must be included in the culture system to preserve spontaneous vascularization in the breast organoids. Co-culturing organoids with endothelial cells (ECs) and fibroblasts in a microfluidic device is one method that has been shown to improve the stemness and survival of organoids. Chemotherapy-induced Notch signaling and VEGF signaling can also improve tumor-derived endothelial microvessels in breast cancer [212]. Fibroblasts can promote vessel development and enhance organoid survival in the side channels of microfluidic systems. Growth factors such as fibroblast growth factor 2 (FGF2) and epidermal growth factor (EGF) can also control multi-lineage differentiation potential, organoid development, and mammosphere regeneration [213]. In stem cells (SCs) and cancer stem cells (CSCs), it has been discovered that FGF2 and EGF either favorably or negatively regulate these activities. Spontaneous vascularization in breast organoids can be preserved by enhancing the culture environment and adding vasculature [214].

#### Vascular organoid on chip model

In biomedical engineering, 3D vascularized microtissues within microfabricated devices have rapidly emerged, and can better replicate tissue microphysiological activity and accurately replicate human diseases in vitro [215]. In the vascular organoid on-chip model, organoids are cultured in a central chamber, and endothelial cells (ECs) and fibroblasts are cultured in the hydrogel in adjacent chambers that are connected to the center chamber, leading to organoid development [216].

Vascular breast organoids were created using a PDMS chip [217]. The PDMS chip was used to fabricate hollow tubes with adjustable diameters and wall thicknesses, which closely emulated the morphology and properties of the human blood vessels [215]. he tubes were then functionalized with human umbilical vein endothelial cells (HUVECs) to construct biomimetic blood vessels [218]. These vascular modules have advantages, such as high optical transparency, gas permeability, and tunable elasticity, making them suitable for integrating multiple organoids into a single microfluidic circuitry [219]. This development in the vascularization of organoids-on-a-chip provides a more controllable and favorable design platform for co-culturing different cells and tissue types, overcoming the limitations of traditional organoid culture [220].

Vascular organoid-on-a-chip models for breast organoids incorporate functional vasculature to facilitate organoid growth and maturation. Organoids that lack functioning vasculature experience necrosis in the core area due to increased metabolic needs that diffusion alone cannot satisfy [121]. Vascularization in organoids and organoids-on-a-chip has been achieved through various strategies, including engineering in vitro capillary beds and integrating them into microfluidic platforms [219]. The development aims to establish a perfused vasculature throughout the organoids, enabling them to survive and operate by supplying oxygen and nutrients and removing metabolic waste. Advancements in intravital 3D bioprinting have also demonstrated the potential for vascularizing organoids [217, 221]. Overall, the incorporation of functional vasculature in organoid-on-a-chip models is crucial for achieving in vivo-like functionality and enhancing the physiological relevance of breast organoid models.

## Involvement of stromal component and immune cells for breast organoid development

The stroma is an essential part of the breast tissue because it offers a favorable microenvironment that affects mammary cell fate, differentiation, and tissue homeostasis. It is becoming increasingly apparent that stromal interactions are essential for the development of healthy mammary tissue and breast cancer. Mammary epithelial cell behavior, including proliferation, migration, and differentiation, is influenced by cues and signals provided by stromal cells. In organoid culture systems, stromal cells play a significant role in promoting the growth and maintenance of mammary epithelial cells in vitro, allowing for long-term culture and study of breast organoids [222].

Breast organoids contain stromal cells that secrete cytokines, chemokines, and growth factors that control the behavior of mammary epithelial cells. For instance, tumor necrosis factor (TNF-α) and transforming growth factor (TGF-β) produced by stromal cells can affect the invasiveness of breast cancer cells, as can stromal-derived factors (SDF-1), which stimulate the growth of mammary progenitor cells. Furthermore, stromal cells have the potential to produce matrix metalloproteinases (MMPs), such as MMP-2, MMP-7, MMP-9, and MMP-11, which are essential for remodeling the extracellular matrix and facilitating tumor invasion and metastasis.

Within the breast organoids, stromal and mammary epithelial cells interact dynamically and reciprocally. Although stromal cells play a crucial role in the growth and function of mammary epithelial cells, mammary epithelial cells, particularly tumor cells, can also influence the phenotype and behavior of stromal cells in the microenvironment. Reciprocal crosstalk between stromal and epithelial cells greatly aids tumor development, invasion, and metastasis [223].

The immune system is another crucial element in the breast tissue microenvironment. Interactions among immune, stromal, and epithelial cells significantly influence breast organoid growth and cancer development. Involvement of the immune system in breast organoids is essential for research on immune responses, inflammation, and potential immunotherapies for breast cancer [224].

Macrophages are essential elements of the tumor microenvironment (TME) and are important for tumor development. The well-established M1/M2 macrophage paradigm emphasizes the distinct functional polarization states of macrophages in response to TME. Previous studies have shown that M1 macrophages are anti-tumor, whereas tumor-associated macrophages (TAMs), also known as M2-polarized macrophages, are linked to pro-tumorigenic outcomes in cancer [225].

M1 macrophages are classically activated macrophages with proinflammatory and tumoricidal phenotypes. They are induced by inflammatory cytokines such as lipopolysaccharide (LPS) and interferon-gamma (IFN-γ). M1 macrophages are characterized by the production of pro-inflammatory cytokines (like interleukin- 1β, interleukin-6, and tumor necrosis factor-alpha) and reactive oxygen species, which support the immune response against tumors and the phagocytosis of tumor cells. In addition, they participate in antigen presentation, promoting the activation of cytotoxic T cells and adaptive immune responses against cancer cells. In contrast, M2 macrophages exhibit pro-tumorigenic and anti-inflammatory phenotypes. They are brought on by anti-inflammatory cytokines frequently found in the TME, like interleukin-4 (IL-4) and interleukin-13 (IL-13). Growth factors secreted by M2 macrophages, such as transforming growth factor-beta (TGF-β) and vascular endothelial growth factor (VEGF), promote angiogenesis, tumor cell proliferation, and metastasis. Additionally, M2 macrophages participate in immune suppression, impairing the anti-tumor immune response by promoting an immunosuppressive environment [226].

M1 and M2 macrophages significantly affected the rapid development of breast cancer in the context of the breast organoid microenvironment. The protumorigenic properties of M2 macrophages, which are TAMs, are thought to aid tumor growth, invasion, and metastasis. In addition to promoting angiogenesis, which aids in vascularization and nutrient supply to the tumor, they also alter the extracellular matrix to promote tumor cell invasion. The ratio of M1 to M2 macrophages within the TME is dynamic and is influenced by several factors, including cytokines, chemokines, and signals from tumor and stromal cells. The interaction of these factors determines whether macrophages are pro- or anti-tumorigenic (M1 or M2) and affects breast cancer development. To develop targeted therapeutic strategies that can modify macrophage polarization and promote anti-tumor immune responses while limiting tumor-promoting effects, it is essential to understand the role of M1 and M2 macrophages in the breast organoid microenvironment.

Immune cells can be incorporated into 3D structures in organoid culture systems, enabling the study of immune cell behavior and interactions with mammary epithelial cells. For example, immune organoids have been used to study immune conditions and to understand the structures and functions of immune tissues in a setting that closely resembles the in vivo microenvironment [227]. Immune breast organoids are three-dimensional culture models created in vitro from patient-derived tumor cells or cell lines using three-dimensional culture technology and cytokines that encourage breast cancer cell proliferation while preventing their apoptosis. Compared with xenografts, which are made from different species and might not accurately reflect the patient’s condition, these organoids have a structure similar to that of breast tumors in the body and provide a more accurate representation of the disease [228]. Cytokines, tumor necrosis factors, interleukins, and transforming growth factor-β play significant roles in breast cancer development and progression. The increased production of cytokines in the tumor microenvironment influences tumor initiation, angiogenesis, and metastasis. Cytokines, such as IL-1, IL-6, IL-11, and TGF-β, promote cancer cell proliferation and invasion. TGF-β has a dual role, acting as a tumor suppressor in the early stages of BC but later promoting tumor progression. TGF-β also mediates the epithelial-to-mesenchymal transition (EMT), which is linked to BC progression and metastasis [229, 230]. Immune breast organoids help research how the immune system and the tumor microenvironment interact. These interactions are essential for cancer growth and response to therapy. Organoids help researchers to better understand the tumor-immune microenvironment and model the immune response against breast cancer cells. This information can direct the development of innovative immunotherapies and enhance the effectiveness of breast cancer treatments [231]. Although immune breast organoids have shown great promise in the study and treatment of cancer, it is important to remember that they still have limitations. For example, they may not accurately replicate the complexity of the human immune system. The accuracy and usefulness of organoids for precision immuno-oncology applications are continuously improving through ongoing research and technological advancements [232].

Additionally, immune cells in the microenvironment of the breast tissue contribute to both the growth of breast organoids and cancer development. On the one hand, immune cells can encourage the development and spread of tumors by secreting cytokines, chemokines, and growth factors that foster a microenvironment favorable to tumors. In contrast, the activation of cytotoxic T cells and natural killer cells enables immune cells to mount anti-tumor responses and inhibit tumor growth.

Recent studies have highlighted the importance of lymphocyte-stromal cell interactions in autoimmune and inflammatory diseases. These interactions most likely affect the immune response within the organoid and its microenvironment, thereby affecting the development of the breast organoids [233]. Researchers can produce more accurate models of breast tissue that replicate crucial elements of the tumor microenvironment by adding stromal components and immune cells to organoid culture. Owing to this improved representation, the investigation of tumor-stroma crosstalk, immune responses, and drug responses can now be performed in a physiologically relevant setting. Breast organoids can be used to study the interactions between mammary epithelial, stromal, and immune cells, which may provide important information regarding the earliest stages of oncogenic transformation and the particular cell types that give rise to various breast cancer subtypes. Additionally, this study may help identify potential therapeutic targets in the immune and stromal compartments to create new breast cancer treatment plans.

## Conclusion

A significant development in cancer research, specifically in the modeling of breast cancer pathophysiology, is presented in the present article. Traditional 2D cell culture models have limitations in accurately simulating the in vivo tumor microenvironment, which has hampered the development of novel clinical techniques and therapies. The poor reproducibility and *in-vtro/* in vivo corelation motivated the researcher to investigate 3D culture systems that closely resemble the complexity and heterogeneity of human tumors. The possibility to recreate a 3D in vitro breast cancer model without scaffolds, enabling a better understanding of tumor behaviour and drug screening. The potential for translation from in vitro to in vivo has significantly improved with the introduction of scaffold-free 3D models, making them more accurate in representing the tumor microenvironment. We have thoroughly discussed how cellular level manipulation is possible to generate heterocellular breast cancer organoid. The physical and mechanical environments offered by 3D organoid culture systems enable cancer cells to develop metastatic potential and drug resistance similar to that of human tumors. We have critically discussed the application of fibroblast in producing extracellular ECM at the boundary layer of organoid to maintain its structural integrity. The application of HUVEC in middle layer to maintain the vascularization of nutrient and drugs in 3D organoid hypoxic core is thoroughly discussed. We have raised the debt, regarding application of scaffold free 3D organoid on counter of conventional scaffold based organoid development. We have dive into deep to identify the responsible abundant extracellular matrix component in 3D breast cancer organoid that closely resembles the human breast cancer microenvironment and their role in tumor cell invasion, angiogenesis and epithelial to mesenchymal transition. The scaffold-free 3D models presented in this article offer a more applicable platform for studying tumor behaviour, disease progression, and drug responses than conventional extracellular matrix-based 3D organoid culture. This article also highlights the importance of developing precise 3D high throughput disease models to study breast cancer biology and to identify potential therapeutic targets. An essential advancement over earlier techniques is the growth of tumor spheroids under controlled conditions using transient intercellular linkers. With the aid of this innovative technique, researchers can now create mature multicellular tumor spheroids in a shorter time, providing a more effective and reliable platform for researching pathophysiology of breast cancer.

## Data Availability

The datasets used and analyzed during the current study are available from the corresponding author upon reasonable request.

## Abbreviations

3D: Three-dimensional
ECM: Extracellular matrix
EMT: Epithelial to mesenchymal transition
MET: Mesenchymal to epithelial transition
LN1: Laminin-111
ER: Estrogen Receptor
ELP: Elastin-like polypeptides
ELR: Elastin-Like Recombinant
GAG: Glycosaminoglycans
PG: Proteoglycans
HDF: Human Dermal Fibroblasts
MEC: Mammary Epithelial Cells
PLGA: Polylactic-co-glycolic acid
PEG: Polyethylene Glycol
GelMA: Gelatin Methacryloyl
PIC: Polyisocyanide
MGO: Mammary gland organoids
PCL: Polycaprolactone
dECM: Decellularized Extracellular Matrix
SDS: Sodium Dodecyl Sulfate
DAMP: Damage-associated Molecular Patterns
EHS: Engelbreth-holm-swarm
BC: Breast cancer
SWH: Salvador-warts hippo
MST1/2: Mammalian Ste20-like kinase 1/2
LATS1/2: Large tumor suppressor 1/2
YAP: Yes-associated protein
ILK: Integrin-linked kinase
PKA: Protein kinase A
PAK: p21-activated kinase
CTGF: Connective tissue growth factor
HA: Hyaluronic Acid
ARD: Ankyrin repeat domain
RyR: Ryanodine receptor
GEF: Guanyl exchange factor
PH: Pleckstrin homology
RBD: Rho-binding domain
ROK: Rho kinase
MLCP: Myosin light chain phosphatase
PI3K: Phosphatidylinositol 3-kinase
PKB: Protein kinase B
TSC: Tuberous sclerosis complex
DH: Dbl Homology
CH: Calponin Homology
ZF: Zinc Finger
SH: Src Homology
PR: Progesterone Receptor
HIS: Human immune system
CAF: Cancer-associated Fibroblasts
TME: Tumor Microenvironment
IGFBP: Insulin-like growth factor-binding proteins
CTHRC1: Collagen triple helix repeat containing-1
hASC: Human adipose tissue-derived stem cells
CSC: Cancer stem cells
BCSC: Breast cancer stem cells
FGF: Fibroblast growth factor
PAF: Proangiogenic factors
VEGF: Vascular endothelial growth factor
Fn: Fibronectin
myCAF: Myofibroblast-like CAFs
TC: Tumor cell
EC: Endothelial cell
2D: Two-dimensional
LaBP: Laser-assisted bioprinting
DMD: Digital Micromirror Device
MMF: Multimode fiber
PVA: Polyvinyl alcohol
EGF: Epidermal growth factor
SC: Stem cell
HUVEC: Human umbilical vein endothelial cells
TNF: Tumor necrosis factor
TGF: Transforming growth factor
SDF: Stromal-derived factors
MMP: Matrix metalloproteinases
TAM: Tumor-associated macrophages
LPS: Lipopolysaccharide
IFN: Interferon
IL: Interleukin
TAZ: Tafazzin
SAV1: Salvador 1
LARG: Leukemia-associated RhoGEF
mTOR: Mammalian target of rapamycin
TIAM1: T-cell lymphoma invasion and metastasis 1
PLA: Polylactic acid

## References

[1] Nair L, Mukherjee S, Kaur K, Murphy CM, Ravichandiran V, Roy S (2023). Multi compartmental 3D breast cancer disease model–recapitulating tumor complexity in in-vitro. Biochim Biophys Acta gen subj.

[2] Rosenbluth JM, Schackmann RCJ, Gray GK, Selfors LM, Li CMC, Boedicker M, et al. Organoid cultures from normal and cancer-prone human breast tissues preserve complex epithelial lineages. Nat Commun. 2020;11. 10.1038/s41467-020-15548-7 PMC7136203 32249764

[3] Goldhammer N, Kim J, Timmermans-Wielenga V, Petersen OW. Characterization of organoid cultured human breast cancer. Breast Cancer Res. 2019;21. 10.1186/s13058-019-1233-x PMC6907265 31829259

[4] Djomehri SI, Burman B, Gonzalez ME, Takayama S, Kleer CG (2019). A reproducible scaffold-free 3D organoid model to study neoplastic progression in breast cancer. J Cell Commun Signal..

[5] Azimian Zavareh V, Rafiee L, Sheikholeslam M, Shariati L, Vaseghi G, Savoji H (2022). Three-dimensional in vitro models: a promising tool to scale-up breast Cancer research. ACS Biomater Sci Eng Am Chemi Soc..

[6] Velasco V, Shariati SA, Esfandyarpour R (2020). Microtechnology-based methods for organoid models. Microsyst Nanoeng.

[7] Kaur S, Kaur I, Rawal P, Tripathi DM, Vasudevan A (2021). Non-matrigel scaffolds for organoid cultures.

[8] Andrews MG, Kriegstein AR (2022). Challenges of Organoid Research.

[9] Marchini A, Gelain F (2022). Synthetic scaffolds for 3D cell cultures and organoids: applications in regenerative medicine.

[10] Zhao Z, Chen X, Dowbaj AM, Sljukic A, Bratlie K, Lin L, et al. Organoids. Nat Rev Methods Primers. 2022;2. 10.1038/s43586-022-00174-y PMC10270325 37325195

[11] Valdoz JC, Johnson BC, Jacobs DJ, Franks NA, Dodson EL, Sanders C, et al. The ECM: to scaffold, or not to scaffold, that is the question. Int J Mol Sci MDPI. 2021;22. 10.3390/ijms222312690 PMC8657545 34884495

[12] Liu J, Long H, Zeuschner D, Räder AFB, Polacheck WJ, Kessler H, et al. Synthetic extracellular matrices with tailored adhesiveness and degradability support lumen formation during angiogenic sprouting. Nat Commun. 2021;12. 10.1038/s41467-021-23644-5 PMC8184799 34099677

[13] Koorman T, Jansen KA, Khalil A, Haughton PD, Visser D, Rätze MAK (2022). Spatial collagen stiffening promotes collective breast cancer cell invasion by reinforcing extracellular matrix alignment. Oncogene..

[14] Liu K, Mihaila SM, Rowan A, Oosterwijk E, Kouwer PHJ (2019). Synthetic extracellular matrices with nonlinear elasticity regulate cellular organization. Biomacromolecules..

[15] Rijal G, Li W (2016). 3D scaffolds in breast cancer research. Biomaterials.

[16] Campbell JJ, Husmann A, Hume RD, Watson CJ, Cameron RE (2017). Development of three-dimensional collagen scaffolds with controlled architecture for cell migration studies using breast cancer cell lines. Biomaterials..

[17] Abbas Y, Brunel LG, Hollinshead MS, Fernando RC, Gardner L, Duncan I, et al. Generation of a three-dimensional collagen scaffold-based model of the human endometrium. Interface Focus. 2020;10. 10.1098/rsfs.2019.0079 PMC7061944 32194932

[18] Redmond J, McCarthy H, Buchanan P, Levingstone TJ, Dunne NJ. Advances in biofabrication techniques for collagen-based 3D in vitro culture models for breast cancer research. Mater Sci Eng C. 2021;122. 10.1016/j.msec.2021.111944 33641930

[19] Dong C, Lv Y. Application of collagen scaffold in tissue engineering: recent advances and new perspectives. Polymers (Basel) MDPI AG. 2016;8. 10.3390/polym8020042 PMC6432532 30979136

[20] Rousselle P, Scoazec JY (2020). Laminin 332 in cancer: when the extracellular matrix turns signals from cell anchorage to cell movement. Semin Cancer Biol Academic Press..

[21] Smuczek B, Santos EDS, Siqueira AS, JJV P, Freitas VM, Jaeger RG (2017). The laminin-derived peptide C16 regulates GPNMB expression and function in breast cancer. Exp Cell Res..

[22] Furuta S, Ren G, Mao J-H, Bissell MJ. Laminin signals initiate the reciprocal loop that informs breast-specific gene expression and homeostasis by activating NO, p53 and microRNAs. 10.7554/eLife.26148.001. 10.7554/eLife.26148 PMC5862529 29560860

[23] Mohammadpour A, Arjmand S, Lotfi AS, Tavana H, Kabir-Salmani M (2018). Promoting hepatogenic differentiation of human mesenchymal stem cells using a novel laminin-containing gelatin cryogel scaffold. Biochem Biophys Res Commun..

[24] Qiu X, Tan H, Fu D, Zhu Y, Zhang J (2018). Laminin is over expressed in breast cancer and facilitate cancer cell metastasis. J Cancer Res Ther..

[25] Kwon SY, Chae SW, Wilczynski SP, Arain A, Carpenter, Philip M (2012). Laminin 332 expression in breast carcinoma. Appl Immunohistochem Mol Morphol.

[26] Yeo GC, Aghaei-Ghareh-Bolagh B, Brackenreg EP, Hiob MA, Lee P, Weiss AS (2015). Fabricated Elastin. Adv Healthc Mater..

[27] Blanco-Fernandez B, Ibañez-Fonseca A, Orbanic D, Ximenes-Carballo C, Perez-Amodio S, Rodríguez-Cabello JC, et al. Elastin-like Recombinamer hydrogels as platforms for breast Cancer modeling. Biomacromolecules. 2022:24. 10.1021/acs.biomac.2c01080 PMC10565832 36597885

[28] Ryan AJ, O’Brien FJ (2015). Insoluble elastin reduces collagen scaffold stiffness, improves viscoelastic properties, and induces a contractile phenotype in smooth muscle cells. Biomaterials..

[29] Leach JB, Wolinsky JB, Stone PJ, Wong JY (2005). Crosslinked α-elastin biomaterials: towards a processable elastin mimetic scaffold. Acta Biomater..

[30] Hinds MT, Rowe RC, Ren Z, Teach J, Wu PC, Kirkpatrick SJ (2006). Development of a reinforced porcine elastin composite vascular scaffold. J Biomed Mater Res A..

[31] Dalton CJ, Lemmon CA (2021). Fibronectin: molecular structure, fibrillar structure and mechanochemical signaling. Cells.

[32] Parisi L, Toffoli A, Ghezzi B, Mozzoni B, Lumetti S, Macaluso GM (2020). A glance on the role of fibronectin in controlling cell response at biomaterial interface.

[33] Shinde A, Libring S, Alpsoy A, Abdullah A, Schaber JA, Solorio L (2018). Autocrine fibronectin inhibits breast cancer metastasis. Mol Cancer Res..

[34] Franchi M, Piperigkou Z, Karamanos KA, Franchi L, Masola V. Extracellular matrix-mediated breast Cancer cells morphological alterations, invasiveness, and microvesicles/exosomes release. Cells. 2020;9. 10.3390/cells9092031 PMC7564980 32899718

[35] Singh N, Patel K, Navalkar A, Kadu P, Datta D, Chatterjee D, et al. Amyloid fibril-based hydrogels for high-throughput tumor spheroid modeling. 10.1101/2020.12.28.424634.

[36] Ambesi A, Maddali P, McKeown-Longo PJ. Fibronectin functions as a selective agonist for distinct toll-like receptors in triple-negative breast Cancer. Cells. 2022;11. 10.3390/cells11132074 PMC9265717 35805158

[37] Barney LE, et al. Tumor cell–organized fibronectin maintenance of a dormant breast cancer population. Sci Adv. 2020;6:eaaz4157. 10.1126/sciadv.aaz4157. 10.1126/sciadv.aaz4157 PMC7065904 32195352

[38] Clegg J, Koch MK, Thompson EW, Haupt LM, Kalita-de Croft P, Bray LJ. Three-dimensional models as a new frontier for studying the role of proteoglycans in the Normal and malignant breast microenvironment. Front Cell Dev Biol Front Media S.A. 2020;8. 10.3389/fcell.2020.569454 PMC7581852 33163489

[39] Habanjar O, Diab-Assaf M, Caldefie-Chezet F, Delort L (2021). 3D cell culture systems: tumor application, advantages, and disadvantages.

[40] Nikitovic D, Kouvidi K, Voudouri K, Berdiaki A, Karousou E, Passi A (2014). The motile breast cancer phenotype roles of proteoglycans/glycosaminoglycans.

[41] Zhang Y, Tang C, Span PN, Rowan AE, Aalders TW, Schalken JA, et al. Polyisocyanide hydrogels as a tunable platform for mammary gland organoid formation. Adv Sci. 2020;7. 10.1002/advs.202001797 PMC7509700 32999851

[42] Malakpour-Permlid A, Buzzi I, Hegardt C, Johansson F, Oredsson S. Identification of extracellular matrix proteins secreted by human dermal fibroblasts cultured in 3D electrospun scaffolds. Sci Rep. 2021;11. 10.1038/s41598-021-85742-0 PMC7988018 33758206

[43] Kyburz KA, Anseth KS (2015). Synthetic mimics of the extracellular matrix: how simple is complex enough?. Ann Biomed Eng..

[44] Tang RZ, Liu XQ. Biophysical cues of in vitro biomaterials-based artificial extracellular matrix guide cancer cell plasticity. Mater Today Bio. 2023;19. 10.1016/j.mtbio.2023.100607 PMC10027567 36960095

[45] Lee HJ, Mun S, Pham DM, Kim P (2021). Extracellular matrix-based hydrogels to tailoring tumor Organoids.

[46] Weiss MS, Bernabé BP, Shikanov A, Bluver DA, Mui MD, Shin S (2012). The impact of adhesion peptides within hydrogels on the phenotype and signaling of normal and cancerous mammary epithelial cells. Biomaterials..

[47] Brösicke N, Sallouh M, Prior LM, Job A, Weberskirch R, Faissner A (2015). Extracellular matrix glycoprotein-derived synthetic peptides differentially modulate glioma and sarcoma cell migration. Cell Mol Neurobiol..

[48] Sthijns MMJPE, van Blitterswijk CA, LaPointe VLS. Synthetic materials that affect the extracellular matrix via cellular metabolism and responses to a metabolic state. Front Bioeng Biotechnol. 2021;9 10.3389/fbioe.2021.742132 PMC8542861 34708025

[49] Dhandayuthapani B, Yoshida Y, Maekawa T, Kumar DS (2011). Polymeric scaffolds in tissue engineering application: a review.

[50] Bock N, Forouz F, Hipwood L, Clegg J, Jeffery P, Gough M, et al. GelMA, click-chemistry gelatin and bioprinted polyethylene glycol-based hydrogels as 3D ex vivo drug testing platforms for patient-derived breast Cancer Organoids. Pharmaceutics. 2023;15. 10.3390/pharmaceutics15010261 PMC9867511 36678890

[51] Quarta A, Gallo N, Vergara D, Salvatore L, Nobile C, Ragusa A, et al. Investigation on the composition of agarose–collagen i blended hydrogels as matrices for the growth of spheroids from breast cancer cell lines. Pharmaceutics. 2021;13. 10.3390/pharmaceutics13070963 PMC8308953 34206758

[52] Rijal G, Bathula C, Li W. Application of synthetic polymeric scaffolds in breast Cancer 3D tissue cultures and animal tumor models. Int J Biomater. 2017;2017. 10.1155/2017/8074890 PMC5828246 29599800

[53] Heo JH, Kang D, Seo SJ, Jin Y (2022). Engineering the extracellular matrix for organoid culture. Int J Stem Cells..

[54] Ligorio C, Mata A (2023). Synthetic extracellular matrices with function-encoding peptides.

[55] Kamatar A, Gunay G, Acar H (2020). Natural and synthetic biomaterials for engineering multicellular tumor spheroids.

[56] Etayash H, Jiang K, Azmi S, Thundat T, Kaur K. Real-time detection of breast cancer cells using peptide-functionalized microcantilever arrays. Sci Rep. 2015;5. 10.1038/srep13967 PMC4593050 26434765

[57] Terzaki K, Kalloudi E, Mossou E, Mitchell EP, Forsyth VT, Rosseeva E, et al. Mineralized self-assembled peptides on 3D laser-made scaffolds: a new route toward “scaffold on scaffold” hard tissue engineering. Biofabrication. 2013;5. 10.1088/1758-5082/5/4/045002 23988557

[58] Unal AZ, West JL (2020). Synthetic ECM: bioactive synthetic hydrogels for 3D tissue engineering. Bioconjug Chem..

[59] Kumar P, Mangla B, Javed S, Ahsan W, Musyuni P, Sivadasan D (2023). A review of nanomaterials from synthetic and natural molecules for prospective breast cancer nanotherapy.

[60] Buchmann B, Engelbrecht LK, Fernandez P, Hutterer FP, Raich MK, Scheel CH, et al. Mechanical plasticity of collagen directs branch elongation in human mammary gland organoids. Nat Commun. 2021;12. 10.1038/s41467-021-22988-2 PMC8115695 33980857

[61] Campaner E, Zannini A, Santorsola M, Bonazza D, Bottin C, Cancila V (2020). Breast cancer organoids model patient-specific response to drug treatment. Cancers (Basel)..

[62] Goel R, Gulwani D, Upadhyay P, Sarangthem V, Singh TD (2023). Unsung versatility of elastin-like polypeptide inspired spheroid fabrication: a review.

[63] Heinz A (2020). Elastases and elastokines: elastin degradation and its significance in health and disease.

[64] Tamayo-Angorrilla M, López de Andrés J, Jiménez G, Marchal JA (2022). The biomimetic extracellular matrix: a therapeutic tool for breast cancer research.

[65] Chakraborty J, Roy S, Ghosh S (2020). Regulation of decellularized matrix mediated immune response. Biomater Sci.

[66] White LJ, Taylor AJ, Faulk DM, Keane TJ, Saldin LT, Reing JE (2017). The impact of detergents on the tissue decellularization process: a ToF-SIMS study. Acta Biomater..

[67] Li J, Chen X, Hu M, Wei J, Nie M, Chen J (2023). The application of composite scaffold materials based on decellularized vascular matrix in tissue engineering: a review.

[68] Zhang X, Chen X, Hong H, Hu R, Liu J, Liu C (2022). Decellularized extracellular matrix scaffolds: recent trends and emerging strategies in tissue engineering. Bioact Mater..

[69] Wang Z, Sun F, Lu Y, Zhang B, Zhang G, Shi H (2021). Rapid preparation method for preparing tracheal Decellularized scaffolds: vacuum assistance and optimization of DNase I. ACS Omega..

[70] Fermor HL, Russell SL, Williams S, Fisher J, Ingham E. Development and characterisation of a decellularised bovine osteochondral biomaterial for cartilage repair. J Mater Sci Mater Med. 2015;26. 10.1007/s10856-015-5517-0 PMC4412724 25893393

[71] Vavken P, Joshi S, Murray MM (2009). TRITON-X is most effective among three decellularization agents for ACL tissue engineering. J Orthop Res..

[72] Kasravi M, Ahmadi A, Babajani A, Mazloomnejad R, Hatamnejad MR, Shariatzadeh S, et al. Immunogenicity of decellularized extracellular matrix scaffolds: a bottleneck in tissue engineering and regenerative medicine. Biomater Res. 2023;27. 10.1186/s40824-023-00348-z PMC9912640 36759929

[73] Pospelov AD, Kutova OM, Efremov YM, Nekrasova AA, Trushina DB, Gefter SD (2023). Breast cancer cell type and biomechanical properties of decellularized mouse organs drives tumor cell colonization. Cells.

[74] Neishabouri A, Soltani Khaboushan A, Daghigh F, Kajbafzadeh AM, Majidi ZM. Decellularization in tissue engineering and regenerative medicine: evaluation, modification, and application methods. Front Bioeng Biotechnol. 2022;10. 10.3389/fbioe.2022.805299 PMC9081537 35547166

[75] Zhang R, Ma M, Dong G, Yao RR, Li JH, Zheng QD (2017). Increased matrix stiffness promotes tumor progression of residual hepatocellular carcinoma after insufficient heat treatment. Cancer Sci..

[76] Zhang M, Xu C, Wang HZ, Peng YN, Li HO, Zhou YJ, et al. Soft fibrin matrix downregulates DAB2IP to promote Nanog-dependent growth of colon tumor-repopulating cells. Cell Death Dis. 2019;10. 10.1038/s41419-019-1309-7 PMC6377646 30770783

[77] Tasdemir N, Bossart EA, Li Z, Zhu L, Sikora MJ, Levine KM (2018). Comprehensive phenotypic characterization human invasive lobular carcinoma cell lines in 2D and 3D cultures. Cancer Res..

[78] Pan D (2010). The hippo signaling pathway in development and cancer. Dev Cell..

[79] Huang J, Zhang L, Wan D, Zhou L, Zheng S, Lin S, et al. Extracellular matrix and its therapeutic potential for cancer treatment. Signal Transduct Target Ther. 2021;6. 10.1038/s41392-021-00544-0 PMC8062524 33888679

[80] Serrano I, McDonald PC, Lock F, Muller WJ, Dedhar S. Inactivation of the hippo tumour suppressor pathway by integrin-linked kinase. Nat Commun. 2013;4. 10.1038/ncomms3976 PMC3905719 24356468

[81] Shi Q, Boettiger D (2003). A novel mode for integrin-mediated signaling: tethering is required for phosphorylation of FAK Y397. Mol Biol Cell..

[82] Lawson CD, Burridge K (2014). The on-off relationship of rho and Rac during integrin-mediated adhesion and cell migration.

[83] Callus BA, Verhagen AM, Vaux DL (2006). Association of mammalian sterile twenty kinases, Mst1 and Mst2, with hSalvador via C-terminal coiled-coil domains, leads to its stabilization and phosphorylation. FEBS J..

[84] Praskova M, Xia F, Avruch J (2008). MOBKL1A/MOBKL1B phosphorylation by MST1 and MST2 inhibits cell proliferation. Curr Biol..

[85] Zhao B, Ye X, Yu J, Li L, Li W, Li S (2008). TEAD mediates YAP-dependent gene induction and growth control. Genes Dev..

[86] Zhao B, Wei X, Li W, Udan RS, Yang Q, Kim J (2007). Inactivation of YAP oncoprotein by the hippo pathway is involved in cell contact inhibition and tissue growth control. Genes Dev..

[87] Zhou D, Conrad C, Xia F, Park JS, Payer B, Yin Y (2009). Mst1 and Mst2 maintain hepatocyte quiescence and suppress hepatocellular carcinoma development through inactivation of the Yap1 oncogene. Cancer Cell..

[88] Lei Q-Y, Zhang H, Zhao B, Zha Z-Y, Bai F, Pei X-H (2008). TAZ promotes cell proliferation and epithelial-mesenchymal transition and is inhibited by the hippo pathway. Mol Cell Biol..

[89] Maurer LM, Ma W, Mosher DF (2016). Dynamic structure of plasma fibronectin. Crit Rev Biochem Mol Biol..

[90] Stivarou T, Patsavoudi E (2015). Extracellular molecules involved in cancer cell invasion. Cancers (Basel)..

[91] Bae YK, Kim A, Kim MK, Choi JE, Kang SH, Lee SJ (2013). Fibronectin expression in carcinoma cells correlates with tumor aggressiveness and poor clinical outcome in patients with invasive breast cancer. Hum Pathol..

[92] Cunha SR, Mohler PJ (2009). Ankyrin protein networks in membrane formation and stabilization. J Cell Mol Med..

[93] Bourguignon LYW (2008). Hyaluronan-mediated CD44 activation of RhoGTPase signaling and cytoskeleton function promotes tumor progression. Semin Cancer Biol..

[94] Lajoie P, Nabi IR (2010). Lipid rafts, caveolae, and their endocytosis. Int Rev Cell Mol Biol..

[95] Noman M, Aysha J, Ketehouli T, Yang J, Du L, Wang F, et al. Calmodulin binding transcription activators: an interplay between calcium signalling and plant stress tolerance. J Plant Physiol. 2021;256. 10.1016/j.jplph.2020.153327 33302232

[96] Haga RB, Ridley AJ (2016). Rho GTPases: regulation and roles in cancer cell biology. Small GTPases..

[97] Schmidt S, Debant A (2014). Function and regulation of the rho guanine nucleotide exchange factor trio. Small GTPases..

[98] Bros M, Haas K, Moll L, Grabbe S. Rhoa as a key regulator of innate and adaptive immunity. Cells. 2019;8. 10.3390/cells8070733 PMC6678964 31319592

[99] Ricker E, Chowdhury L, Yi W, Pernis AB (2016). The RhoA-ROCK pathway in the regulation of T and B cell responses [version 1; referees: 3 approved]. F1000Res.

[100] Amano M, Nakayama M, Kaibuchi K (2010). Rho-kinase/ROCK: A key regulator of the cytoskeleton and cell polarity. Cytoskeleton..

[101] Julian L, Olson MF (2014). Rho-associated coiled-coil containing kinases (ROCK), structure, regulation, and functions.

[102] Bourguignon LYW (2014). Matrix hyaluronan-activated CD44 signaling promotes keratinocyte activities and improves abnormal epidermal functions. Am J Pathol..

[103] Fruman DA, Chiu H, Hopkins BD, Bagrodia S, Cantley LC, Abraham RT (2017). The PI3K pathway in human disease.

[104] Yang J, Nie J, Ma X, Wei Y, Peng Y, Wei X (2019). Targeting PI3K in cancer: mechanisms and advances in clinical trials.

[105] Cai Z, Zhang F, Chen W, Zhang J, Li H (2019). Mirnas: a promising target in the chemoresistance of bladder cancer.

[106] Chen CY, Chen J, He L, Stiles BL (2018). PTEN: tumor suppressor and metabolic regulator. Front Endocrinol (Lausanne).

[107] Worby CA, Dixon JE (2014). Pten. Annu Rev Biochem.

[108] Levina A, Fleming KD, Burke JE, Leonard TA. Activation of the essential kinase PDK1 by phosphoinositide-driven trans-autophosphorylation. Nat Commun. 2022:13. 10.1038/s41467-022-29368-4 PMC8986801 35387990

[109] Kim S, Heo S, Brzostowski J, Kang D. Endosomal mtorc2 is required for phosphoinositide-dependent akt activation in platelet-derived growth factor-stimulated glioma cells. Cancers (Basel). 2021;13. 10.3390/cancers13102405 PMC8157044 34065746

[110] Dangelmaier C, Manne BK, Liverani E, Jin J, Bray P, Kunapuli SP (2013). PDK1 selectively phosphorylates Thr(308) on Akt and contributes to human platelet functional responses. Thromb Haemost..

[111] Nitulescu GM, Van De Venter M, Nitulescu G, Ungurianu A, Juzenas P, Peng Q (2018). The Akt pathway in oncology therapy and beyond (review). Int J Oncol..

[112] He Y, Sun MM, Zhang GG, Yang J, Chen KS, Xu WW, et al. Targeting PI3K/Akt signal transduction for cancer therapy. Signal Transduct Target Ther. 2021;6. 10.1038/s41392-021-00828-5 PMC8677728 34916492

[113] Kotelevets L, Chastre E (2020). Rac1 signaling: from intestinal homeostasis to colorectal cancer metastasis.

[114] Kowluru A (2017). Tiam1/Vav2-Rac1 axis: A tug-of-war between islet function and dysfunction.

[115] Xu Z, Gakhar L, Bain FE, Spies M, Fuentes EJ (2017). The Tiam1 guanine nucleotide exchange factor is autoinhibited by its pleckstrin homology coiled-coil extension domain. J Biol Chem..

[116] Jiang Y, Prabakaran I, Wan F (2014). Vav2 protein overexpression marks and may predict the aggressive subtype of ductal carcinoma in situ. Biomark Res.

[117] Zhang Y, Yang X, Liu Y, Ge L, Wang J, Sun X, et al. Vav2 is a novel APP-interacting protein that regulates APP protein level. Sci Rep. 2022;12. 10.1038/s41598-022-16883-z PMC9325707 35882892

[118] Carvalho AM, Reis RL, Pashkuleva I. Hyaluronan receptors as mediators and modulators of the tumor microenvironment. Adv Healthc Mater. 2023;12. 10.1002/adhm.202202118 PMC11469756 36373221

[119] Białkowska K, Komorowski P, Bryszewska M, Miłowska K (2020). Spheroids as a type of three-dimensional cell cultures—examples of methods of preparation and the most important application. Int J Mol Sci MDPI AG..

[120] Chae SJ, Hong J, Hwangbo H, Kim GH (2021). The utility of biomedical scaffolds laden with spheroids in various tissue engineering applications. Theranostics..

[121] Srivastava V, Huycke TR, Phong KT, Gartner ZJ. Organoid models for mammary gland dynamics and breast cancer. Curr Opin Cell Biol. 2020;66:51–8. 10.1016/j.ceb.2020.05.003. 10.1016/j.ceb.2020.05.003 PMC8175015 32535255

[122] Chen G, Liu W, Yan B (2022). Breast Cancer MCF-7 cell spheroid culture for drug discovery and development. J Cancer Ther..

[123] Kang YP, Yoon JH, Long NP, Koo GB, Noh HJ, Oh SJ, et al. Spheroid-induced epithelial-mesenchymal transition provokes global alterations of breast cancer lipidome: a multi-layered omics analysis. Front Oncol. 2019;9. 10.3389/fonc.2019.00145 PMC6437068 30949448

[124] Santos SJ, Aupperlee MD, Xie J, Durairaj S, Miksicek R, Conrad SE (2009). Progesterone receptor A-regulated gene expression in mammary organoid cultures. J Steroid Biochem Mol Biol..

[125] Luyckx V, Durant JF, Camboni A, Gilliaux S, Amorim CA, Van Langendonckt A (2013). Is transplantation of cryopreserved ovarian tissue from patients with advanced-stage breast cancer safe? A pilot study. J Assist Reprod Genet..

[126] Tian H, Lyu Y, Yang YG, Hu Z (2020). Humanized rodent models for Cancer research.

[127] Onaciu A, Munteanu R, Munteanu VC, Gulei D, Raduly L, Feder RI (2020). Spontaneous and induced animal models for Cancer research.

[128] Dekkers JF, van Vliet EJ, Sachs N, Rosenbluth JM, Kopper O, Rebel HG (2021). Long-term culture, genetic manipulation and xenotransplantation of human normal and breast cancer organoids. Nat Protoc..

[129] Guillen KP, Fujita M, Butterfield AJ, Scherer SD, Bailey MH, Chu Z (2022). A human breast cancer-derived xenograft and organoid platform for drug discovery and precision oncology. Nat Cancer..

[130] Dunpall R, Opoku AR, Revaprasadu N (2016). Development and characterization of MCF7 mammary carcinoma xenografts in a non-immunocompromised rat model. Trop J Pharm Res..

[131] De Souza N (2018). Organoids. Nat methods.

[132] Nawy T. Capturing microbial interactions. Nat Methods Nat Res. 2017;35.

[133] Kozlowski MT, Crook CJ, Ku HT. Towards organoid culture without Matrigel. Commun Biol. 2021;4 10.1038/s42003-021-02910-8 PMC8664924 34893703

[134] Li C, Teixeira AF, Zhu HJ, ten Dijke P. Cancer associated-fibroblast-derived exosomes in cancer progression. Mol Cancer. 2021;20. 10.1186/s12943-021-01463-y PMC8638446 34852849

[135] Mao X, Xu J, Wang W, Liang C, Hua J, Liu J, et al. Crosstalk between cancer-associated fibroblasts and immune cells in the tumor microenvironment: new findings and future perspectives. Mol Cancer. 2021;20. 10.1186/s12943-021-01428-1 PMC8504100 34635121

[136] Hosein AN, Livingstone J, Buchanan M, Reid JF, Hallett M, Basik M. A functional in vitro model of heterotypic interactions reveals a role for interferon-positive carcinoma associated fibroblasts in breast cancer. BMC Cancer. 2015;15. 10.1186/s12885-015-1117-0 PMC4369836 25884794

[137] Weigel KJ, Jakimenko A, Conti BA, Chapman SE, Kaliney WJ, Leevy WM (2014). CAF-secreted IGFBPs regulate breast cancer cell anoikis. Mol Cancer Res..

[138] Li H, Liu W, Zhang X, Wang YF. Cancer-associated fibroblast-secreted collagen triple helix repeat containing-1 promotes breast cancer cell migration, invasiveness and epithelial-mesenchymal transition by activating the Wnt/β-catenin pathway. Oncol Lett. 2021;22. 10.3892/ol.2021.13075 PMC8503808 34671428

[139] Millet M, Bollmann E, Ringuette Goulet C, Bernard G, Chabaud S, Huot MÉ, et al. Cancer-associated fibroblasts in a 3D engineered tissue model induce tumor-like matrix stiffening and EMT transition. Cancers (Basel). 2022;14. 10.3390/cancers14153810 PMC9367573 35954473

[140] Poon S, Ailles LE. Modeling the role of Cancer-associated fibroblasts in tumor cell invasion. Cancers (Basel). 2022;14. 10.3390/cancers14040962 PMC8870277 35205707

[141] Akkouch A, Yu Y, Ozbolat IT. Microfabrication of scaffold-free tissue strands for three-dimensional tissue engineering. Biofabrication. 2015;7. 10.1088/1758-5090/7/3/031002 26373778

[142] Chawla S, Chameettachal S, Ghosh S (2015). Probing the role of scaffold dimensionality and media composition on matrix production and phenotype of fibroblasts. Mater Sci Eng C..

[143] Franchi-Mendes T, Eduardo R, Domenici G, Brito C. 3D cancer models: depicting cellular crosstalk within the tumour microenvironment. Cancers (Basel) MDPI. 2021;13. 10.3390/cancers13184610 PMC8468887 34572836

[144] Guo W (2014). Concise review: breast Cancer stem cells: regulatory networks, stem cell niches, and disease relevance. Stem Cells Transl Med..

[145] Crabtree JS, Miele L (2018). Breast cancer stem cells.

[146] Sasmita AO, Wong YP. Organoids as reliable breast Cancer study models: an update. Int J Oncol Res. 2018;2018.

[147] Bahcecioglu G, Basara G, Ellis BW, Ren X, Zorlutuna P (2020). Breast cancer models: Engineering the tumor microenvironment. Acta Biomater..

[148] Fiorini E, Veghini L, Corbo V. Modeling cell communication in Cancer with Organoids: making the complex simple. Front Cell Dev Biol Frontiers Media SA. 2020;8. 10.3389/fcell.2020.00166 PMC7094029 32258040

[149] De Pieri A, Rochev Y, Zeugolis DI. Scaffold-free cell-based tissue engineering therapies: advances, shortfalls and forecast. NPJ Regen Med Nat Res. 2021;6. 10.1038/s41536-021-00133-3 PMC8007731 33782415

[150] Irie Y, Mizumoto H, Fujino S, Kajiwara T (2008). Reconstruction of cartilage tissue using scaffold-free organoid culture technique. J Biosci Bioeng..

[151] Blache U, Horton ER, Xia T, Schoof EM, Blicher LH, Schönenberger A, et al. Mesenchymal stromal cell activation by breast cancer secretomes in bioengineered 3D microenvironments. Life Sci Alliance. 2019;2. 10.26508/lsa.201900304 PMC6549139 31160380

[152] Baskaran JP, Weldy A, Guarin J, Munoz G, Kotlik M, Subbiah N, et al. Cell shape, and not 2D migration, predicts ECM-driven 3D cell invasion in breast cancer. 10.1101/2019.12.31.892091. 10.1063/1.5143779 PMC7202897 32455252

[153] Keller F, Rudolf R, Hafner M (2019). Towards optimized breast cancer 3D spheroid mono-and co-culture models for pharmacological research and screening. J Cell Biotechnol..

[154] Shekhar MPV, Pauley R, Heppner G (2003). Extracellular matrix-stromal cell contribution to neoplastic phenotype of epithelial cells in the breast. Breast Cancer Res..

[155] Swaminathan S, Ngo O, Basehore S, Clyne AM (2017). Vascular endothelial-breast epithelial cell Coculture model created from 3D cell structures. ACS Biomater Sci Eng..

[156] Kay EJ, Paterson K, Riero-Domingo C, Sumpton D, Däbritz JHM, Tardito S (2022). Cancer-associated fibroblasts require proline synthesis by PYCR1 for the deposition of pro-tumorigenic extracellular matrix. Nat Metab..

[157] Mohan SC, Lee TY, Giuliano AE, Cui X (2021). Current status of breast organoid models.

[158] Franchi-Mendes T, Lopes N, Brito C. Heterotypic tumor spheroids in agitation-based cultures: a scaffold-free cell model that sustains Long-term survival of endothelial cells. Front Bioeng Biotechnol. 2021;9. 10.3389/fbioe.2021.649949 PMC8219978 34178955

[159] Shoval H, Karsch-Bluman A, Brill-Karniely Y, Stern T, Zamir G, Hubert A, et al. Tumor cells and their crosstalk with endothelial cells in 3D spheroids. Sci Rep. 2017;7. 10.1038/s41598-017-10699-y PMC5585367 28874803

[160] Yu T, Di G (2017). Role of tumor microenvironment in triple-negative breast cancer and its prognostic significance.

[161] Guarneri V, Conte P (2009). Metastatic breast Cancer: therapeutic options according to molecular subtypes and Prior adjuvant therapy. Oncologist..

[162] Harbeck N, Penault-Llorca F, Cortes J, Gnant M, Houssami N, Poortmans P, et al. Breast cancer. Nat Rev Dis Primers. 2019;5. 10.1038/s41572-019-0111-2 31548545

[163] Loibl S, Gianni L (2017). HER2-positive breast cancer. The lancet.

[164] Yoder R, Kimler BF, Staley JM, Schwensen K, Wang YY, Finke K, et al. Impact of low versus negative estrogen/progesterone receptor status on clinico-pathologic characteristics and survival outcomes in HER2-negative breast cancer. NPJ Breast Cancer. 2022;8. 10.1038/s41523-022-00448-4 PMC9273627 35817765

[165] Welch DR (2016). Tumor heterogeneity - a “contemporary concept” founded on historical insights and predictions. Cancer res.

[166] Aazmi A, Zhang D, Mazzaglia C, Yu M, Wang Z, Yang H (2024). Biofabrication methods for reconstructing extracellular matrix mimetics.

[167] Tan Q, Xu L, Zhang J, Ning L, Jiang Y, He T (2023). Breast cancer cells interact with tumor-derived extracellular matrix in a molecular subtype-specific manner. Biomater Adv..

[168] Rafaeva M, Jensen ARD, Horton ER, Zornhagen KW, Strøbech JE, Fleischhauer L, et al. Fibroblast-derived matrix models desmoplastic properties and forms a prognostic signature in cancer progression. Front Immunol. 2023;14. 10.3389/fimmu.2023.1154528 PMC10395327 37539058

[169] Byrne CE, Decombe J-B, Bingham GC, Remont J, Miller LG, Khalif L (2021). Evaluation of extracellular matrix composition to improve breast Cancer modeling. Tissue Eng Regen Med Int Soc..

[170] Caruso M, Huang S, Mourao L, Scheele CLGJ. A mammary organoid model to study branching morphogenesis. Front Physiol. 2022;13. 10.3389/fphys.2022.826107 PMC8988230 35399282

[171] Cui J, Guo W. Establishment and long-term culture of mouse mammary stem cell organoids and breast tumor organoids. STAR Protoc. 2021;2. 10.1016/j.xpro.2021.100577 PMC8173303 34124696

[172] Padmanaban V, Grasset EM, Neumann NM, Fraser AK, Henriet E, Matsui W (2020). Organotypic culture assays for murine and human primary and metastatic-site tumors. Nat Protoc..

[173] Duarte AA, Gogola E, Sachs N, Barazas M, Annunziato S, Ruiter RDJ (2018). BRCA-deficient mouse mammary tumor organoids to study cancer-drug resistance. Nat Methods..

[174] Yip HYK, Papa A. Generation and functional characterization of murine mammary organoids. STAR Protoc. 2021;2. 10.1016/j.xpro.2021.100765 PMC8403683 34485937

[175] Strobel HA, Moss SM, Hoying JB (2023). Vascularized tissue Organoids.

[176] Li X, Zhu D (2023). Advances in breast cancer organoid for individualized treatment. Organs-on-a-Chip..

[177] Shi W, Mirza S, Kuss M, Liu B, Hartin A, Wan S (2023). Embedded bioprinting of breast tumor cells and Organoids using low-concentration collagen-based bioinks.

[178] Li Y, Khuu N, Prince E, Tao H, Zhang N, Chen Z (2021). Matrix stiffness-regulated growth of breast tumor spheroids and their response to chemotherapy. Biomacromolecules..

[179] Kinstlinger IS, Calderon GA, Royse MK, Means AK, Grigoryan B, Miller JS (2021). Perfusion and endothelialization of engineered tissues with patterned vascular networks. Nat Protoc Nat Res.

[180] Yu J (2021). Vascularized Organoids: a more complete model. Int J Stem Cells..

[181] Xu H, Jiao D, Liu A, Wu K (2022). Tumor organoids: applications in cancer modeling and potentials in precision medicine.

[182] Lancaster MA, Knoblich JA. Organogenesisin a dish: Modeling development and disease using organoid technologies. Sci Am Assoc Adv Sci. 2014;345. 10.1126/science.1247125 25035496

[183] Zhang W, Wray LS, Rnjak-Kovacina J, Xu L, Zou D, Wang S (2015). Vascularization of hollow channel-modified porous silk scaffolds with endothelial cells for tissue regeneration. Biomaterials..

[184] Mansour AA, Gonçalves JT, Bloyd CW, Li H, Fernandes S, Quang D (2018). An in vivo model of functional and vascularized human brain organoids. Nat Biotechnol..

[185] Nashimoto Y, Hayashi T, Kunita I, Nakamasu A, Torisawa YS, Nakayama M (2017). Integrating perfusable vascular networks with a three-dimensional tissue in a microfluidic device. Integr Biol (United Kingdom)..

[186] Liew AWL, Zhang Y (2017). In vitro pre-vascularization strategies for tissue engineered constructs-bioprinting and others. Int J Bioprint..

[187] Dababneh AB, Ozbolat IT. Bioprinting technology: a current state-of-the-art review. J Manuf Sci Eng, Trans ASME. 2014;136.

[188] Gungor-Ozkerim PS, Inci I, Zhang YS, Khademhosseini A, Dokmeci MR (2018). Bioinks for 3D bioprinting: an overview. Biomater Sci.

[189] Wang Z, Abdulla R, Parker B, Samanipour R, Ghosh S, Kim K. A simple and high-resolution stereolithography-based 3D bioprinting system using visible light crosslinkable bioinks. Biofabrication. 2015;7. 10.1088/1758-5090/7/4/045009 26696527

[190] Donderwinkel I, Van Hest JCM, Cameron NR (2017). Bio-inks for 3D bioprinting: recent advances and future prospects. Polym Chem Royal Soc Chemistry.

[191] Khoeini R, Nosrati H, Akbarzadeh A, Eftekhari A, Kavetskyy T, Khalilov R (2021). Natural and synthetic bioinks for 3D bioprinting. Adv Nanobiomed Res..

[192] Ramiah P, du Toit LC, Choonara YE, Kondiah PPD, Pillay V (2020). Hydrogel-based bioinks for 3D bioprinting in tissue regeneration.

[193] Mir A, Lee E, Shih W, Koljaka S, Wang A, Jorgensen C (2023). 3D bioprinting for vascularization.

[194] Anthon SG, Valente KP (2022). Vascularization strategies in 3D cell culture models: from scaffold-free models to 3D bioprinting.

[195] Rawal P, Tripathi DM, Ramakrishna S, Kaur S (2021). Prospects for 3D bioprinting of organoids.

[196] Ren Y, Yang X, Ma Z, Sun X, Zhang Y, Li W (2021). Developments and opportunities for 3D bioprinted Organoids.

[197] Reid JA, Palmer XL, Mollica PA, Northam N, Sachs PC, Bruno RD. A 3D bioprinter platform for mechanistic analysis of tumoroids and chimeric mammary organoids. Sci Rep. 2019;9. 10.1038/s41598-019-43922-z PMC6522494 31097753

[198] Mollica PA, Booth-Creech EN, Reid JA, Zamponi M, Sullivan SM, Palmer XL (2019). 3D bioprinted mammary organoids and tumoroids in human mammary derived ECM hydrogels. Acta Biomater..

[199] Ma X, Kato Y, Kempen F, Hirai Y, Tsuchiya T, Keulen F (2015). Multiple patterning with process optimization method for maskless DMD-based grayscale lithography.

[200] Allen J (2017). Application of patterned illumination using a DMD for optogenetic control of signaling. Nat Methods..

[201] Grebenyuk S, Ranga A (2019). Engineering organoid vascularization.

[202] Nayak B, Balachander GM, Manjunath S, Rangarajan A, Chatterjee K (2019). Tissue mimetic 3D scaffold for breast tumor-derived organoid culture toward personalized chemotherapy. Colloids Surf B Biointerfaces..

[203] Huo CW, Huang D, Chew GL, Hill P, Vohora A, Ingman WV (2016). Human glandular organoid formation in murine engineering chambers after collagenase digestion and flow cytometry isolation of normal human breast tissue single cells. Cell Biol Int..

[204] Nagarajan S, Belaid H, Radhakrishnan S, Teyssier C, Balme S, Miele P, et al. Sacrificial mold-assisted 3D printing of stable biocompatible gelatin scaffolds. Bioprinting. 2021;22.

[205] Liu S, Wang T, Li S, Wang X (2022). Application status of sacrificial biomaterials in 3D bioprinting.

[206] Li S, Li H, Shang X, He J, Hu Y. Recent advances in 3D printing sacrificial templates for fabricating engineered vasculature. MedComm – Biomater Appl [Internet]. 2023;2 Available from: 10.1002/mba2.46 .

[207] Gergely RCR, Pety SJ, Krull BP, Patrick JF, Doan TQ, Coppola AM (2015). Multidimensional vascularized polymers using degradable sacrificial templates. Adv Funct Mater..

[208] Tseng TC, Hsieh FY, Theato P, Wei Y, Hsu S, hui. (2017). Glucose-sensitive self-healing hydrogel as sacrificial materials to fabricate vascularized constructs. Biomaterials..

[209] Brassard JA, Lutolf MP (2019). Engineering stem cell self-organization to build better Organoids.

[210] Athanasiou KA, Eswaramoorthy R, Hadidi P, Hu JC (2013). Self-organization and the self-assembling process in tissue engineering. Annu Rev Biomed Eng..

[211] Liu H, Zhang X, Liu J, Qin J (2023). Vascularization of engineered organoids.

[212] Zhang P, He D, Chen Z, Pan Q, Du F, Zang X (2016). Chemotherapy enhances tumor vascularization via notch signaling-mediated formation of tumor-derived endothelium in breast cancer. Biochem Pharmacol..

[213] Merikhian P, Darvishi B, Jalili N, Esmailinejad MR, Khatibi AS, Kalbolandi SM (2022). Recombinant nanobody against MUC1 tandem repeats inhibits growth, invasion, metastasis, and vascularization of spontaneous mouse mammary tumors. Mol Oncol..

[214] Schulla LS, Alupoaie ED, De Silva L, Gawlitta D, Middendorp S, Coffer PJ, et al. Development of a novel microfluidic co-culture model to study organoid vascularization. 10.1101/2022.03.25.485813.

[215] Zhang S, Wan Z, Kamm RD (2021). Vascularized organoids on a chip: strategies for engineering organoids with functional vasculature.

[216] Gunti S, Hoke ATK, Vu KP, London NR (2021). Organoid and spheroid tumor models: techniques and applications.

[217] Shirure VS, Hughes CCW, George SC (2021). Engineering Vascularized Organoid-on-a-Chip Models.

[218] Zhang W, Zhang YS, Bakht SM, Aleman J, Shin SR, Yue K (2016). Elastomeric free-form blood vessels for interconnecting organs on chip systems. Lab Chip..

[219] Zhao X, Xu Z, Xiao L, Shi T, Xiao H, Wang Y, et al. Review on the vascularization of Organoids and Organoids-on-a-Chip. Front Bioeng Biotechnol. 2021;9. 10.3389/fbioe.2021.637048 PMC8072266 33912545

[220] Lim J, Ching H, Yoon JK, Jeon NL, Kim YT (2021). Microvascularized tumor organoids-on-chips: advancing preclinical drug screening with pathophysiological relevance.

[221] Song K, Zu X, Du Z, Hu Z, Wang J, Li J. Diversity models and applications of 3d breast tumor-on-a-chip. Micromachines (Basel). 2021;12. 10.3390/mi12070814 PMC8306159 34357224

[222] Cerchiari AE, Garbe JC, Jee NY, Todhunter ME, Broaders KE, Peehl DM (2015). A strategy for tissue self-organization that is robust to cellular heterogeneity and plasticity. Proc Natl Acad Sci U S A..

[223] Dykes SS, Hughes VS, Wiggins JM, Fasanya HO, Tanaka M, Siemann D. Stromal cells in breast cancer as a potential therapeutic target [internet]. Oncotarget. 2018; Available from: www.oncotarget.com. 10.18632/oncotarget.25245 PMC5955086 29805773

[224] Bar-Ephraim YE, Kretzschmar K, Clevers H (2020). Organoids in immunological research. Nat Rev Immunol Nat Res..

[225] Boutilier AJ, Elsawa SF. Macrophage polarization states in the tumor microenvironment. Int J Mol Sci MDPI. 2021;22. 10.3390/ijms22136995 PMC8268869 34209703

[226] Liu J, Geng X, Hou J, Wu G (2021). New insights into M1/M2 macrophages: key modulators in cancer progression.

[227] Ye W, Luo C, Li C, Huang J, Liu F (2020). Organoids to study immune functions, immunological diseases and immunotherapy.

[228] Yu J, Huang W (2020). The Progress and clinical application of breast Cancer Organoids. Int J Stem Cells..

[229] Esquivel-Velázquez M, Ostoa-Saloma P, Palacios-Arreola MI, Nava-Castro KE, Castro JI, Morales-Montor J (2015). The role of cytokines in breast cancer development and progression. J Interferon Cytokine Res Mary Ann Liebert Inc..

[230] Méndez-García LA, Nava-Castro KE, Ochoa-Mercado TDL, Palacios-Arreola MI, Ruiz-Manzano RA, Segovia-Mendoza M (2019). Breast Cancer metastasis: are cytokines important players during its development and progression?. J Interferon Cytokine Res.

[231] Sun CP, Lan HR, Fang XL, Yang XY, Jin KT. Organoid models for precision Cancer immunotherapy. Front Immunol Frontiers Media SA. 2022;13. 10.3389/fimmu.2022.770465 PMC9016193 35450073

[232] Zhao J, Fong A, Seow SV, Toh HC (2023). Organoids as an enabler of precision Immuno-oncology.

[233] Koning JJ, Mebius RE (2020). Stromal cells and immune cells involved in formation of lymph nodes and their niches.

